# Characterization of a novel OTX2‐driven stem cell program in Group 3 and Group 4 medulloblastoma

**DOI:** 10.1002/1878-0261.12177

**Published:** 2018-03-01

**Authors:** Margaret Stromecki, Nazanin Tatari, Ludivine Coudière Morrison, Ravinder Kaur, Jamie Zagozewski, Gareth Palidwor, Vijay Ramaswamy, Patryk Skowron, Matthias Wölfl, Till Milde, Marc R. Del Bigio, Michael D. Taylor, Tamra E. Werbowetski‐Ogilvie

**Affiliations:** ^1^ Regenerative Medicine Program Department of Biochemistry and Medical Genetics University of Manitoba Winnipeg Canada; ^2^ Ottawa Bioinformatics Core Facility Ottawa Hospital Research Institute Canada; ^3^ The Arthur and Sonia Labatt Brain Tumour Research Center The Hospital for Sick Children Toronto Canada; ^4^ Division of Haematology/Oncology University of Toronto and The Hospital for Sick Children Canada; ^5^ Program in Neuroscience and Mental Health and Division of Neurology The Hospital for Sick Children Toronto Canada; ^6^ Arthur and Sonia Labatt Brain Tumour Research Centre and Program in Developmental and Stem Cell Biology The Hospital for Sick Children Toronto Canada; ^7^ University Children's Hospital Pediatric Oncology, Hematology and Stem Cell Transplantation University of Würzburg Germany; ^8^ Center for Individualized Pediatric Oncology (ZIPO) and Brain Tumors Translational Program Hopp‐Children's Cancer Center at the NCT (KiTZ) Heidelberg Germany; ^9^ CCU Pediatric Oncology (G340) German Cancer Research Center (DKFZ) and German Consortium for Translational Cancer Research (DKTK) Heidelberg Germany; ^10^ Department of Pathology University of Manitoba and The Children's Hospital Research Institute of Manitoba Winnipeg Canada

**Keywords:** axon guidance genes, medulloblastoma, orthodenticle homeobox 2, RHO, semaphorin, stem cells

## Abstract

Medulloblastoma (MB) is the most common malignant primary pediatric brain cancer. Among the most aggressive subtypes, Group 3 and Group 4 originate from stem/progenitor cells, frequently metastasize, and often display the worst prognosis, yet we know the least about the molecular mechanisms driving their progression. Here, we show that the transcription factor orthodenticle homeobox 2 (OTX2) promotes self‐renewal while inhibiting differentiation *in vitro* and increases tumor initiation from MB stem/progenitor cells *in vivo*. To determine how OTX2 contributes to these processes, we employed complementary bioinformatic approaches to characterize the OTX2 regulatory network and identified novel relationships between OTX2 and genes associated with neuronal differentiation and axon guidance signaling in Group 3 and Group 4 MB stem/progenitor cells. In particular, OTX2 levels were negatively correlated with semaphorin (SEMA) signaling, as expression of 9 *SEMA* pathway genes is upregulated following OTX2 knockdown with some being potential direct OTX2 targets. Importantly, this negative correlation was also observed in patient samples, with lower expression of *SEMA4D* associated with poor outcome specifically in Group 4 tumors. Functional proof‐of‐principle studies demonstrated that increased levels of select SEMA pathway genes are associated with decreased self‐renewal and growth *in vitro* and *in vivo* and that RHO signaling, known to mediate the effects of SEMA genes, is contributing to the OTX2 KD phenotype. Our study provides mechanistic insight into the networks controlled by OTX2 in MB stem/progenitor cells and reveals novel roles for axon guidance genes and their downstream effectors as putative tumor suppressors in MB.

**Abbreviations**
MB: medulloblastoma
SEMA: semaphorin

## 1 Introduction

Medulloblastoma (MB) is currently divided into at least five molecular subgroups that exhibit different genomic aberrations, gene expression profiles, and clinical outcomes as well as extensive intertumoral heterogeneity: WNT, Sonic Hedgehog (SHH)/*TP53*‐wild‐type, SHH/*TP53* mutant, Group 3, and Group 4 (Cavalli *et al*., 2017; Louis *et al*., 2016; Northcott *et al*., 2011). Indeed, recent studies have identified up to 12 MB subtypes within the subgroups that display unique genetic, epigenetic and molecular signatures (Cavalli *et al*., 2017; Northcott *et al*., 2017; Schwalbe *et al*., 2017). High‐risk to very‐high‐risk patients include those exhibiting the more aggressive Group 3 and Group 4 MB subgroups (Ramaswamy *et al*., 2016). These children have the worst prognosis, with up to 50% displaying metastatic dissemination through the cerebrospinal fluid at diagnosis (Ramaswamy *et al*., 2016). Metastases are attributed to persistent cancer stem cells and highly motile cells that evade chemotherapy and radiation treatment. However, surprisingly little is known about the genes and signaling pathways that regulate these treatment‐resistant cell populations. Thus, there is a critical need to identify the pathways contributing to Group 3 and Group 4 MB pathogenesis not only to understand how these tumors progress but also to develop targeted therapies with less harmful side‐effects on the developing brains of children. As these tumors originate from stem/progenitor cells that exist transiently during early cerebellar development (Kawauchi *et al*., 2012, 2017; Lin *et al*., 2016; Pei *et al*., 2012), targeting MB cells with stem/progenitor molecular signatures that persist beyond this stage represents a therapeutic strategy that may have less toxic effects on the nervous systems of young patients.

The homeodomain transcription factor orthodenticle homeobox 2 (OTX2) plays critical roles in forebrain, midbrain, and rostral hindbrain patterning as well as lineage specification (Acampora *et al*., 1995; Ang *et al*., 1996; Matsuo *et al*., 1995). Through its homeodomain, the OTX2 protein binds to the DNA target sequence 5′‐TAATCC‐3′ with high affinity (Beby and Lamonerie, 2013). OTX2 activates or suppresses its targets both directly and indirectly through additional intermediate signaling molecules or other transcription factors (Bai *et al*., 2012; Bunt *et al*., 2012). For example, OTX2 enhances proliferation of ventral midbrain progenitor cells (Omodei *et al*., 2008), while it inhibits proliferation in the thalamus (Puelles *et al*., 2006), demonstrating that OTX2 function is dependent on both neuroanatomical region and cell type. While expression is tightly controlled during normal neurodevelopment, OTX2 levels are abnormally sustained in Group 3 and Group 4 MB, with over 80% exhibiting either recurrent gain or overexpression (OE) of this homeobox gene (Adamson *et al*., 2010).

To date, most studies have evaluated the role of OTX2 specifically on MB cell proliferation and survival (Bunt *et al*., 2010, 2012). Experiments involving OTX2 OE or knockdown in established MB cell lines grown as adherent cultures in serum have identified cell cycle genes as direct targets of OTX2 (Bunt *et al*., 2010, 2012). However, Bunt *et al*. (2013) suggested that OTX2 may not actually regulate gene expression levels directly and demonstrated that OTX2 functions to sustain H3K27 trimethylation and maintain promoter bivalency. While these studies provided significant insight on how OTX2 contributes to MB growth, a more comprehensive understanding of OTX2‐mediated regulation in stem cell conditions will be critical, as cancer stem cells are major contributors to tumor initiation, recurrence, and poor prognosis in Group 3 and Group 4 MB patients.

We have recently discovered a novel role for OTX2 in controlling stem cell function or self‐renewal in established Group 3 and Group 4 MB cell lines (Kaur *et al*., 2015). However, the mechanisms by which OTX2 regulates this process were not defined. Here, we characterized an OTX2‐driven stem cell program in Group 3 and Group 4 MB stem/progenitor cells and identified a negative correlation between OTX2 and expression of a large cohort of axon guidance genes. Specifically, OTX2 was negatively correlated with nine semaphorin ligands and receptors. Functional studies demonstrated that increased levels of genes involved in semaphorin signaling and the downstream RHO pathway are associated with a more differentiated phenotype. Our data reveal novel associations between OTX2 and axon guidance genes and underscore a potential tumor‐suppressive role for these neurodevelopmental cues in Group 3 and Group 4 tumors.

## 2 Materials and methods

### 2.1 Cell culture

D283 and D341 cells were purchased from the American Type Culture Collection (ATCC, Rockville, MD, USA). D283 (Friedman *et al*., 1985) exhibits features of both Group 3 (Thompson *et al*., 2017) and Group 4 MB (Snuderl *et al*., 2013), and D341 is a Group 3 MB cell line (Friedman *et al*., 1988). D425 (He *et al*., 1991) Group 3 cells were obtained from Magimairajan Vanan (University of Manitoba, Winnipeg, MB, Canada). D283 cells were cultured as previously described (Kaur *et al*., 2015). D341 and D425 cells were maintained in StemPro^®^ Neural Stem Cell Serum Free Medium (Life Technologies, Burlington, ON, Canada) on ultra‐low‐attachment plates. MB3W1 (Dietl *et al*., 2016) and HD‐MB03 (Milde *et al*., 2012) Group 3 cells were cultured as previously described. Confluent cultures were dissociated in Accutase (Life Technologies) and passed 1 : 10 for maintenance (Kaur *et al*., 2015). All cell lines were recently authenticated by STR profiling (ATCC) and maintained for a maximum of 20–25 passages before a new vial was thawed. For tumorsphere assays, D283 and MB3W1 cells were dissociated and plated at 1–20 cells·μL^−1^ onto 24‐well ultra‐low‐attachment plates in Neural Stem Cell Media (Kaur *et al*., 2015) and Stem Cell Media consisting of Dulbecco's modified Eagle's medium/F12 supplemented with 1% N2 (Gibco, Burlington, ON, Canada), 1% B27 (Gibco), 20 ng·mL^−1^ epidermal growth factor (R&D Systems, Minneapolis, MN, USA), 20 ng·mL^−1^ basic fibroblast growth factor (bFGF; R&D Systems), and 4% penicillin/streptomycin 10 000 U·mL^−1^ (Life Technologies), respectively. D341, D425, and HD‐MB03 cells were cultured in StemPro media. For SEMA protein treatment, human L1CAM (R&D Systems), SEMA4D (R&D Systems), and NRP1 (ACRO Biosystems, Newark, DE, USA) Fc chimera proteins were added to D283 tumorsphere cultures (50–1000 ng·mL^−1^) at day 0 for both primary and secondary passage.

### 2.2 Small interfering RNA

OTX2, SEMA4D, L1CAM, and NRP1 levels were knocked down in MB cells using 30 nm Silencer select siRNA (Life Technologies), while a nonsilencing (scramble) siRNA was used as a negative control. OTX2 was knocked down using three independent siRNA sequences (s9931, s9932, and s9933), while the SEMA genes were knocked down using two independent siRNA sequences for SEMA4D (s51388 and s20598), L1CAM (s8036, s8038), and NRP1 (s16843, s16844). Knockdown was evaluated by immunoblot.

### 2.3 Gene expression profiling and analyses

Extracted RNA from D283 scramble control (OTX2^high^) and OTX2 KD (OTX2^low^) tumorspheres using siRNA 9931 (*N* = 3 biological replicates) was subjected to GeneChip 3′ oligonucleotide microarray hybridization and processing performed by Stem Core Laboratories at the Ottawa Hospital Research Institute (OHRI). Analysis was performed by the Ottawa Bioinformatics Core Facility. HuGene 2.0 st microarray RMA expression values were generated by the Affymetrix Expression Console. Gene symbol annotations were based on Affymetrix provided HuGene‐2_0‐st‐v1.na35.hg19 transcript cluster annotations, assigning each transcript cluster identifier to the first provided gene symbol in the annotation. Fold change and significance for transcript cluster identifiers between conditions were determined using the R limma package. Differentially expressed pathways were analyzed using ingenuity pathway analysis (IPA; Redwood City, CA, USA). Transcripts differentially expressed at least twofold (up‐ or downregulated) and with a value of *P* < 0.05 were considered significant. Downstream effects analysis was conducted using the *Z*‐score algorithm to predict the expected causal effects between differentially expressed genes and cell function. A *Z*‐score ≥ 2 indicates that the function is significantly increased, whereas a *Z*‐score ≤ −2 indicates that the function is significantly decreased. Gene set enrichment analysis (GSEA) (Subramanian *et al*., 2005) was performed on expression data comparing OTX2^high^ and OTX2^low^ tumorspheres (Affymetrix HuGene 2.0st). GSEA results were explored using the Reactome (Croft *et al*., 2014) and KEGG (Kanehisa *et al*., 2016) databases to identify pathways significantly enriched in the expression sets.

### 2.4 Chromatin immunoprecipitation sequencing

Chromatin immunoprecipitation was performed on D283 tumorspheres as previously described (Kaur *et al*., 2015). ChIP‐seq data were generated on a NextSeq 2500 and mapped to the GRCh38 human genome model using bowtie2 v2.2.4. Peaks were called using macs2 v2.1.0.20140616. Peaks called were masked for ENCODE blacklist peak locations.

### 2.5 Immunofluorescent staining

Tumorspheres were fixed in 10% formalin for 2–3 h. Samples were washed 2× with PBS and incubated in fresh, ice‐cold 15% sucrose followed by 30% sucrose each for 2–3 h at 4 °C. Samples were embedded in OCT, frozen, cut into 10‐μm‐thick sections, washed in Tris‐buffered saline (TBS 1×) for 10 min, and incubated with 1% BSA and 5% serum in TBS for 45 min at room temperature (RT). Sections were stained with mouse antineuron‐specific βIII‐tubulin monoclonal antibody (1 : 100) (R&D Systems) and incubated at RT for 2 h. Slides were then washed with TBS 3 × 5 min, followed by secondary antibody incubation (Alexa flour 488 goat anti‐mouse IgG) (1 : 200) (ThermoFisher) for 1 h at RT. Slides were then washed and counterstained with prolong gold antifade mountant with DAPI (Molecular Probes, Life Technologies, Eugene, OR, USA).

### 2.6 Immunoblotting

Protein was isolated using either the All‐In‐One Purification Kit (Norgen Biotek, Thorold, ON, Canada) or using IP lysis buffer (975 μl lysis buffer, 10 μl 50× protease inhibitor, and 5 μl orthovanadate). Twenty micrograms of protein was loaded onto 10% Tris/glycine gels. Nitrocellulose membranes were blocked in 2.5% nonfat milk in TBS with Tween‐20 (TBST) and then incubated at 4°C overnight with antibodies diluted in SuperSignal antibody buffer at concentrations described in Table S1. Membranes were treated with enhancer buffer for 10 min prior to blocking with 2.5% nonfat milk. Membranes were washed 3× with TBST before application of secondary antibodies (Table S1) and then developed using SuperSignal West Pico (Fisher Scientific, Nepean, ON, Canada).

### 2.7 Real‐time qPCR

Total RNA was extracted from tumorspheres, and first‐strand cDNA was synthesized as previously described (Kaur *et al*., 2015). GoTaq qPCR Master Mix (Fisher Scientific) was used for sample preparation, and analysis was performed on a Mx3000P (Stratagene, Santa Clara, CA, USA) system. Glyceraldehyde 3‐phosphate dehydrogenase (*GAPDH*) was used to normalize all values. Primer sequences are listed in Table S2. The following qPCR conditions were used: 50 °C for 2 min, 95 °C for 2 min, and 40 cycles of 95 °C for 15 s and 60 °C for 30 s.

### 2.8 Lentiviral transduction

Stable knockdown of OTX2 was performed as previously described (Kaur *et al*., 2015). Stable SEMA4D OE was performed using ORF cDNA lentiviral particles (GeneCopoeia, Rockville, MD, USA) consisting of an Lv105 transfer vector with a puromycin resistance gene. Lentiviral negative control particles were used as controls. Cells were seeded in six‐well plates at 2 × 10^5^ in stem cell conditions 24 h prior to transduction.

### 2.9 RHO pull‐down

RHO activity was measured using the Active RHO Pull‐Down and Detection kit according to manufacturer's instructions (ThermoFisher). For each treatment, 12–18 wells of tumorspheres were washed with ice‐cold TBS and resuspended in 0.5 ml lysis buffer (25 mm Tris/HCl, pH 7.2, 150 mm NaCl, 5 mm MgCl_2_, 1% NP‐40 and 5% glycerol, and 10 μl 50× protease inhibitor). Samples were evaluated for RHO activity by immunoblot.

### 2.10 Group 3 and Group 4 MB Patient sample analysis

Fully annotated Affymetrix Gene 1.1 ST Array datasets (Northcott *et al*., 2012; Remke *et al*., 2011) were used to compare transcript levels of axon guidance genes in 234 Group 3 and Group 4 patient samples. The following axon guidance genes were not part of this published microarray dataset: *GNG3*,* EFNA4*,* ARPC1B*,* TUBB4A*,* MIR27B*, and *ITGA4*. A Pearson correlation coefficient was calculated for OTX2 and each of the axon guidance genes. The FDR correction was used to adjust for multiple comparisons (FDR < 0.1). Survival was compared in patients with SEMA gene expression (> 80th percentile) to patients with low SEMA gene expression (< 20th percentile), and the associated *P*‐value was calculated.

Survival was also assessed across 377 primary Group 3 and Group 4 MB samples, profiled on the Affymetrix Gene 1.1 ST array as previously described, normalized using the RMA method, but subgrouped using similarity network fusion (GSE85217) (Cavalli *et al*., 2017). Overall survival was analyzed by the Kaplan–Meier method, and *P*‐values were reported using the log‐rank test. The Cox's proportional hazards model for survival‐time (time‐to‐event) outcomes was calculated using log2‐transformed gene expression as the predictor. All statistical analyses were performed in the r statistical environment (v3.3.3), using r packages survival (v2.40‐1), and ggplot2 (v2.2.1).

### 2.11 Intracerebellar transplantation

The University of Manitoba Animal Care Committee approved all procedures. Dissociated tumorspheres from D283 scramble and OTX2 KD or D283 control and D283 SEMA4D OE tumorspheres were injected into the cerebellum of 5‐ to 7‐week‐old NOD‐SCID mice. Animals were anesthetized and injected with 2 × 10^5^, 1 × 10^5^, or 5 × 10^4^ MB cells. When animals reached endpoint after 40–45 days, brains were perfused and samples extracted and prepared for histopathological analysis as previously described (Kaur *et al*., 2015).

For immunohistochemical staining, antigen retrieval was performed by boiling in citrate buffer (pH 6.0) for 20 min followed by a 30 min cool down. Samples were blocked for 1 h at RT with 10% sheep serum in PBS. Primary antibody (Ki67, Cell Signaling Technology, Danvers, MA, USA; 1 : 800 or SEMA4D, Sigma‐Aldrich, Oakville, ON, Canada; 1 : 75) in 1% sheep serum in PBS was applied overnight at 4 °C. Samples were then incubated for 2 h at RT in secondary biotin anti‐mouse or anti‐rabbit antibody (Jackson ImmunoResearch, West Grove, PA, USA; 1 : 500). Streptavidin antibody (Jackson ImmunoResearch, 1 : 400) was applied for 30 min at RT followed by 2 min (Ki67) or 30 s (SEMA4D) in DAB. Samples were counterstained, dehydrated, and mounted with Permount.

### 2.12 Statistical tests

Statistical analyses were performed using prism 5 software (GraphPad Software, La Jolla, CA, USA). One‐way ANOVAs and Tukey's tests for multiple comparisons were employed for OTX2 siRNA tumorsphere data. Tumor data were analyzed using an independent sample one‐tailed *t*‐test with Welch's correction. Recombinant Fc protein tumorsphere data and dual OTX2/SEMA gene KD data were analyzed using a one‐way ANOVA followed by a Dunnett's test for multiple comparisons. Normalized ROCK inhibitor data were analyzed using a two‐way ANOVA followed by Dunnett's test. All data are reported as a mean ± standard error of the mean (SEM). A *P*‐value less than 0.05 was considered significant.

## 3 Results

### 3.1 OTX2 knockdown decreases MB self‐renewal while increasing differentiation from tumorspheres *in vitro*

**Figure 1:**
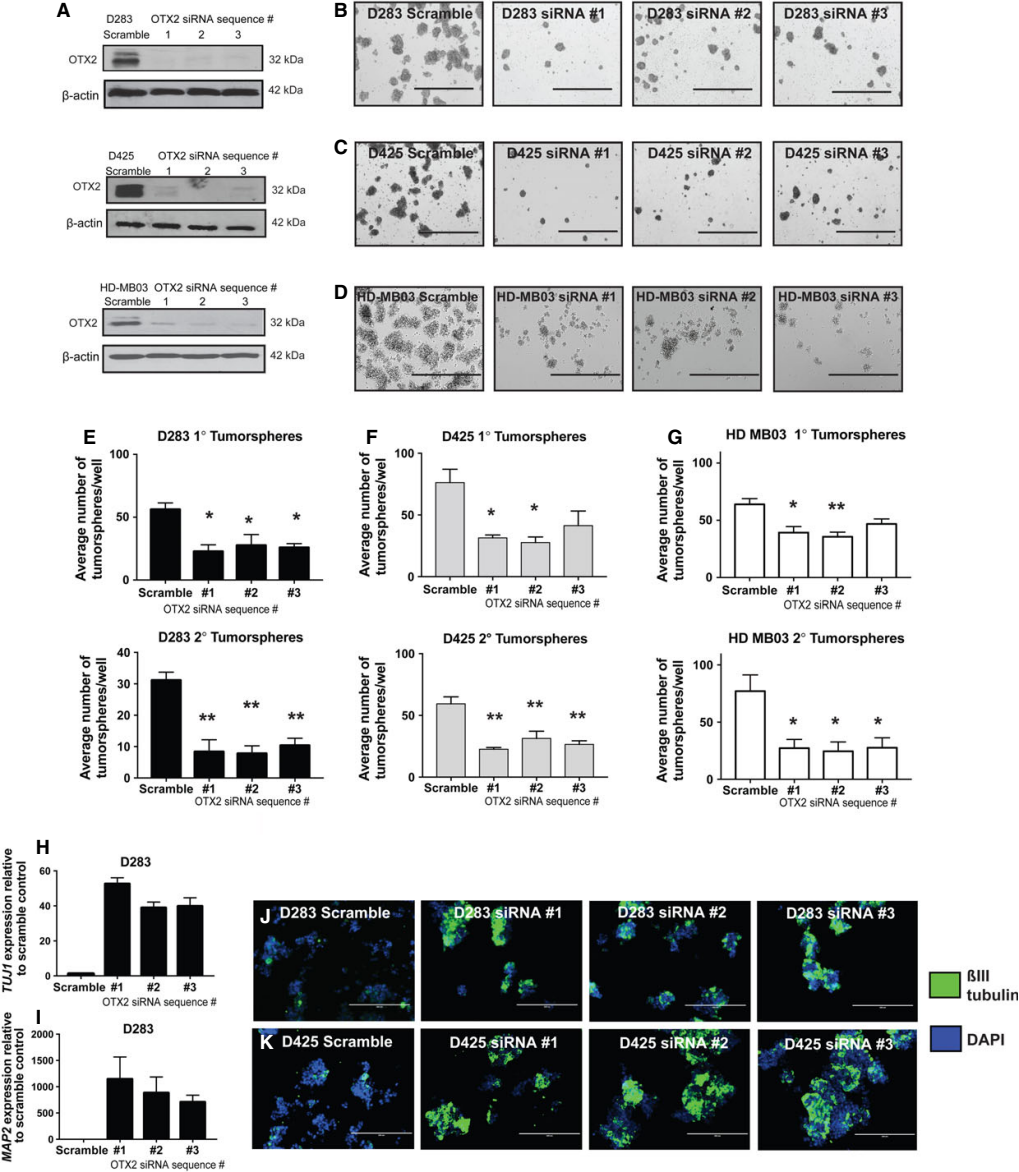
Knockdown of OTX2 in Group 3 and Group 4 MB tumorspheres decreases self‐renewal and increases differentiation. (A) Immunoblot validation of OTX2 knockdown in tumorspheres from the D283 and the D425 MB cell lines as well as the recently derived HD‐MB03 cell line using three independent siRNA sequences relative to scramble siRNA. β‐Actin serves as a loading control. (B–D) Representative images of tumorspheres at secondary passage following OTX2 knockdown in D283 (B), D425 (C), and HD‐MB03 (D) cells. Scale bar: 1000 μm. (E–G) Quantification of primary (upper) and secondary (lower) tumorsphere number in D283 (E) D425 (F) and HD‐MB03 (G) tumorspheres following OTX2 knockdown. Error bars: SEM. *P* < 0.05*, *P* < 0.01**. For all experiments, *N* = 3 or *N* = 4 biological replicates or independent transfections for each siRNA. (H, I) *TUJ1* (βIII‐tubulin) (H) and *MAP2* (I) expression following OTX2 knockdown using three siRNA sequences in D283 tumorspheres by qPCR. Error bars: SEM. *N* = 3 biological replicates. (J, K) Immunofluorescent staining of D283 (J) and D425 (K) tumorspheres for βIII‐tubulin following OTX2 knockdown using three siRNA sequences. Scale bar: 200 μm.

We previously showed that OTX2 knockdown (KD) decreases self‐renewal capacity in the D283 and D341 cell lines (Kaur *et al*., 2015). To further validate this effect on self‐renewal, we knocked down OTX2 in D283, D425, and D341 Group 3 tumorspheres as well as the recently established HD‐MB03 (Milde *et al*., 2012) and MB3W1 (Dietl *et al*., 2016) Group 3 primary cell lines (Fig. 1A, Fig. S1A,E). OTX2 KD in all 5 cell lines resulted in a significant decrease in tumorsphere formation and self‐renewal capacity (Fig. 1B–G, Fig. S1B–D, F–H). We previously demonstrated that OTX2 KD decreases cell growth and viability in tumorsphere culture (Kaur *et al*., 2015); however, we did not determine whether these effects were also accompanied by increased differentiation. To explore this, we evaluated the expression of neuronal differentiation markers following OTX2 KD by qPCR and immunofluorescence staining (IF). *TUJI* (βIII‐tubulin) and *MAP2* transcript levels were strongly upregulated in D283 tumorspheres following OTX2 KD (Fig. 1H,I). Similarly, βIII‐tubulin was upregulated by IF in D283 and D425 tumorspheres following OTX2 KD (Fig. 1J,K). These results extend our previous findings in established cell lines and reveal that OTX2 KD also decreases self‐renewal capacity in recently derived Group 3 MB cells while concomitantly increasing neuronal differentiation. Thus, OTX2 is important for regulating the balance between self‐renewal and differentiation in MB cells.

### 3.2 OTX2 knockdown decreases growth and tumor‐initiating capacity *in vivo*

**Figure 2:**
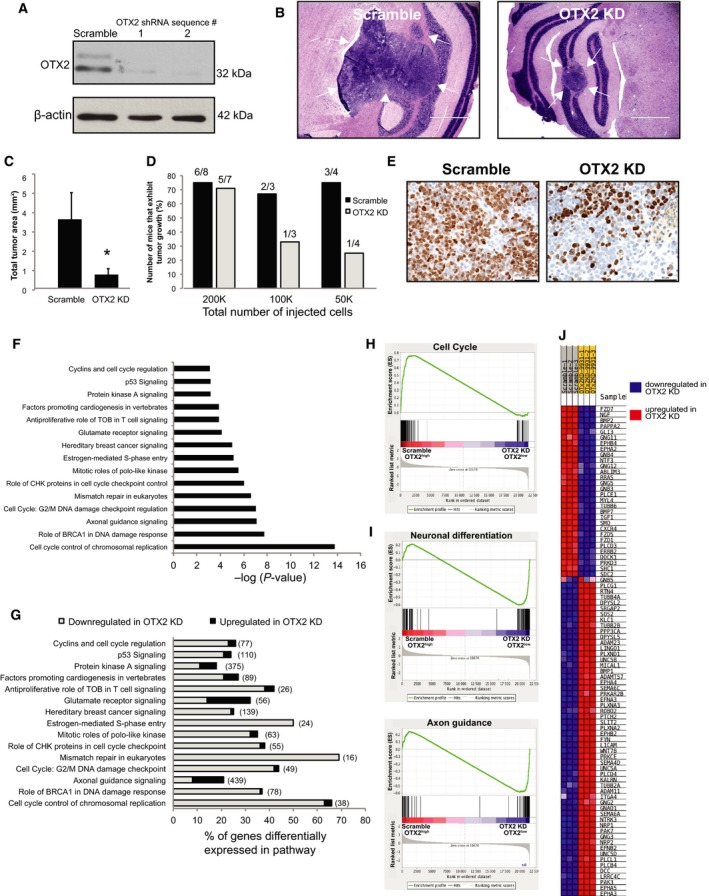
OTX2 knockdown decreases tumor growth and tumor‐initiating capacity *in vivo* and increases levels of neuronal differentiation and axon guidance genes. (A) Immunoblot validation of stable OTX2 knockdown in D283 cells using two shRNA sequences relative to scramble control. β‐Actin serves as a loading control. (B) Representative images of tumors derived from D283 scramble or D283 OTX2 knockdown cells following injection into the cerebellum of NOD SCID mice. Scale bar: 1000 μm. Arrows denote intracerebellar tumors from each. (C) Quantification of tumor area following intracerebellar injection of 2 × 10^5^ D283 scramble (*N* = 8) or OTX2 knockdown (*N* = 7) tumorsphere cells. Error bars: SEM. *P* < 0.05*. (D) Limiting dilution analysis of tumors derived from D283 scramble relative to D283 OTX2 knockdown tumorspheres following intracerebellar injection. OTX2 knockdown cells exhibit a decrease in tumor‐initiating capacity as indicated by the lower number of animals displaying evidence of tumor growth. Scale bar: 1000 μm. (E) Representative images of Ki67 staining in tumors derived from D283 scramble or D283 OTX2 knockdown cells following injection into NOD SCID mice. Scale bar: 50 μm. (F,G) IPA analysis showing major pathways (F) affected by OTX2 knockdown in D283 tumorspheres and the frequency of genes within these pathways (G) that are upregulated (black) and downregulated (gray). (H) Gene set enrichment analysis (GSEA) demonstrating enrichment of genes associated with cell cycle in the control scramble D283 tumorspheres. (I) GSEA demonstrating that neuronal differentiation and axon guidance genes are enriched in genes sets that are downregulated in the scramble and upregulated in the OTX2 KD D283 tumorspheres. (J) Representative heat map of genes that are significantly downregulated (blue) and upregulated (red) following OTX2 knockdown in D283 tumorspheres. Note that the majority of genes are upregulated following OTX2 knockdown.

We next evaluated the effects of OTX2 KD on tumor growth and tumor‐initiating capacity, a feature associated with cancer stem cell function (Clarke *et al*., 2006; de Sousa e Melo *et al*., 2017), *in vivo*. We previously generated stable D283 OTX2 KD cells using two shRNA sequences (Kaur *et al*., 2015). Stable OTX2 KD results in a significant decrease in tumorsphere number and cell growth, albeit to a lesser extent than the OTX2 KD siRNA (Kaur *et al*., 2015). Thus, we are able to expand the cells just enough to utilize for *in vivo* studies. Here, we generated new stable OTX2 KD cells using the same validated two shRNA sequences (Fig. 2A) and injected 2 × 10^5^ cells derived from tumorspheres for both D283 scramble (*N* = 8) and D283 OTX2 KD (sequence #2) cells (*N* = 7) into the cerebellum of NOD‐SCID mice. Tumors derived from OTX2 KD tumorspheres (0.7 ± 0.3 mm^2^) were significantly smaller than those derived from scramble controls (3.6 ± 1.4 mm^2^) (Fig. 2B,C). Importantly, limiting dilution analysis comparing tumor growth from 2 × 10^5^, 1 × 10^5^, and 5 × 10^4^ D283 scramble relative to D283 OTX2 KD tumorsphere cells revealed a decrease in tumor‐initiating capacity following OTX2 KD (Fig. 2D). Only small nests of tumor cells were observed, if any, following injection of 5 × 10^4^ D283 OTX2 KD cells (Fig. 2D). This was supported by a decrease in Ki67 staining for tumor cell proliferation in D283 OTX2 KD tumors (Fig. 2E). Taken together, these results demonstrate that OTX2 contributes to both tumor growth and tumor initiation from MB tumorspheres *in vivo*.

### 3.3 OTX2 expression is negatively correlated with the majority of axon guidance genes in MB

We next sought to evaluate the molecular mechanisms by which OTX2 regulates MB stem/progenitor cells. We performed global gene expression analysis using Human Gene 2.0 microarrays to compare the molecular profiles of D283 OTX2‐expressing (OTX2^high^) scramble tumorspheres relative to OTX2 KD (OTX2low) tumorspheres generated by siRNA (Fig. 1A). Of the 3614 significantly and differentially (*P* < 0.05 and ±2fold) expressed transcripts in OTX2^high^ relative to OTX2^low^ tumorspheres, pathways associated with cell cycle and neuronal differentiation/axon guidance, including ephrin, netrin, slit, and semaphorin (SEMA) signaling, represented the top dysregulated networks (Fig. 2F,G; Tables S3 and S4). This was further supported by GSEA that demonstrated an enrichment of genes associated with cell cycle in the OTX2^high^ tumorspheres (Fig. 2H), whereas enrichment of genes associated with neuronal differentiation and axon guidance (Fig. 2I) was observed in the OTX2^low^ tumorspheres. IPA downstream effects analysis confirmed these findings with 85 of 252 differentially expressed neural development genes exhibiting measurement directions consistent with an increase in neuronal differentiation (*Z*‐score: 3.2) (Table S3). Of the 90 axon guidance gene transcripts that were significantly and differentially expressed, 57 (or 63%) were upregulated following OTX2 KD (Fig. 2J, Table S4).

To determine whether axon guidance genes are directly or indirectly regulated by OTX2, chromatin immunoprecipitation (ChIP) sequencing (ChIP‐Seq) was performed on OTX2^high^ D283 tumorspheres. A clear, statistically significant association was observed between differential expression of axon guidance genes and the presence of one or more OTX2 binding peaks within −5 kb to +2 kb of the transcription start site (TSS) for each gene (Fisher's exact test; *P* < 2.2e‐16) (Table S5). Semaphorin (SEMA) signaling was the most overrepresented pathway. SEMA genes are classically known as inhibitory axon growth cone guidance cues, but have also been found to play prominent roles in tumor cell proliferation, survival, cell adhesion, angiogenesis, and migration in other cancers (Neufeld *et al*., 2016). The SEMA ligands are membrane‐bound or secreted proteins that mediate their effects mainly through plexin (PLXN) receptors with neuropilins (NRPs) often serving as coreceptors (Neufeld *et al*., 2016). The transmembrane protein L1CAM interacts with neuropilin 1 (NRP1) and is also a coreceptor (Neufeld *et al*., 2016). Of the nine SEMA ligands or receptors negatively correlated with OTX2 expression, five genes exhibited OTX2 overlaps/binding peaks and two genes (*NRP1* and *SEMA6C*) display OTX2‐binding motifs (TAATCT and/or TAATCC) within the region of the TSS. As expression of all SEMA pathway genes was upregulated following OTX2 KD, our results suggest that OTX2 may serve as a direct or indirect repressor of SEMA signaling.

### 3.4 Axon guidance genes are negatively correlated with OTX2 expression in MB tumorspheres as well as Group 3 and Group 4 patient samples

We next validated the negative correlation between OTX2 and SEMA gene expression at the transcript and/or protein levels in MB cell lines as well as Group 3 and Group 4 patient samples. Select SEMA pathway genes, including the SEMA ligands (*SEMA4D*,* SEMA6A*) and receptors (*NRP1*,* L1CAM*,* PLXNA2*) (Fig. S2A–C) as well as several members of other axon guidance gene families (Fig. S3A,B), were evaluated in OTX2^high^ scramble and OTX2^low^ (OTX2 KD) MB tumorspheres by qPCR. In all cell lines, OTX2 expression was negatively correlated with all axon guidance gene transcript levels (Figs S2 and S3).

**Figure 3:**
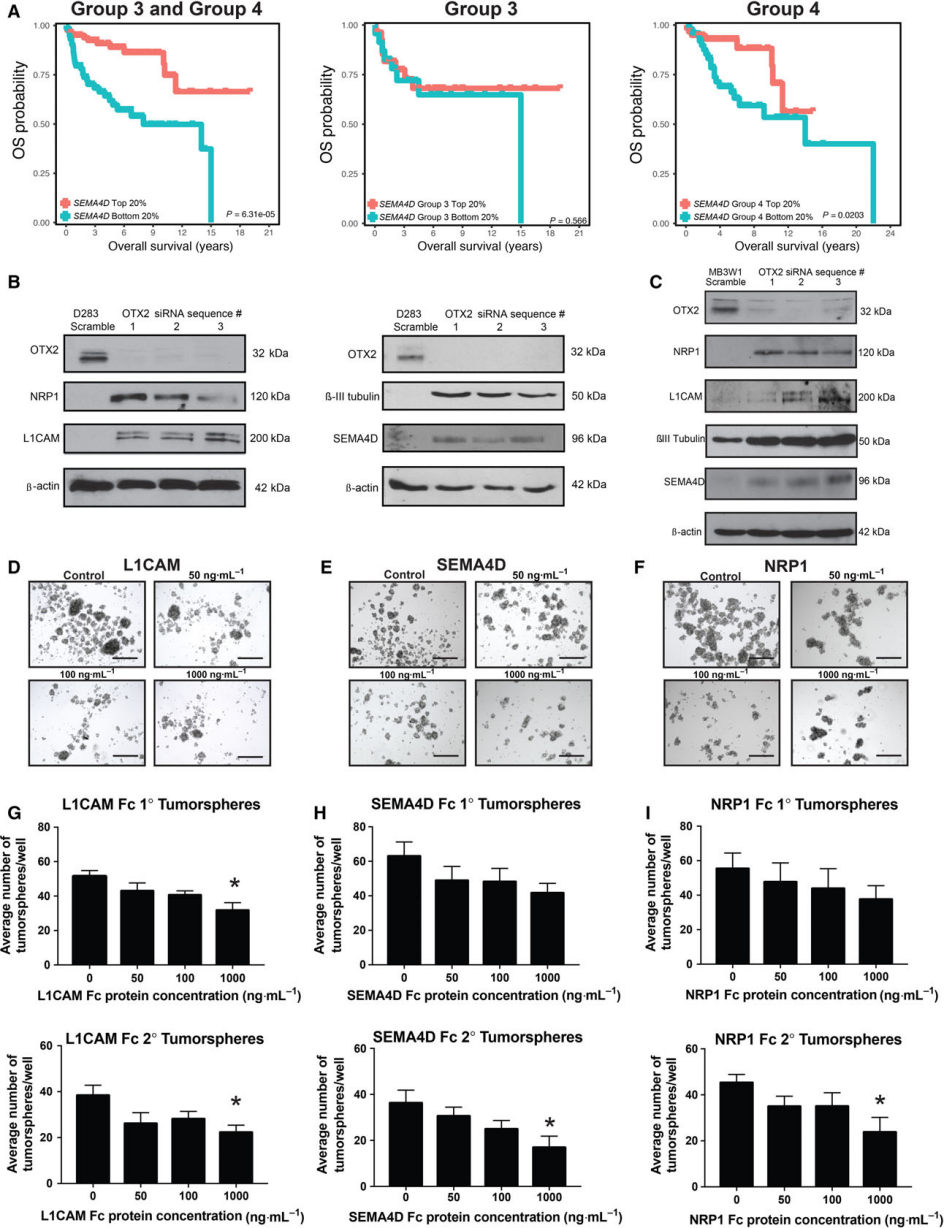
Semaphorin genes are negatively correlated with OTX2 expression and self‐renewal in MB tumorspheres. (A) Kaplan–Meier curves of Group 3 and Group 4 patients with high (red) and low (blue) *SEMA4D* expression for Group 3 and Group 4 combined (left), Group 3 alone (middle), and Group 4 alone (right). (B) Immunoblots depicting increases in NRP1, L1CAM, PLXNA2, SEMA4D, and βIII‐tubulin protein levels following OTX2 knockdown in D283 tumorspheres. β‐Actin serves as a loading control. (C) Immunoblots depicting increases in NRP1, L1CAM, SEMA4D, and βIII‐tubulin protein levels following OTX2 knockdown in MB3W1 tumorspheres using three siRNA sequences. β‐Actin serves as a loading control. (D–F) Representative images of D283 tumorspheres following 5‐day treatment with recombinant L1CAM Fc (D), SEMA4D Fc (E), or NRP1 Fc (F) chimera protein. *N* = 4 biological replicates. (G–I) Total number of primary (upper) and secondary (lower) tumorspheres following 5‐day treatment with L1CAM Fc (G) SEMA4D Fc (H) or NRP1 Fc chimera protein (I). *N* = 4 biological replicates. Error bars: SEM. *P* < 0.05*. Scale bar: 400 μm.

Importantly, significant correlations between OTX2 and axon guidance pathway genes were also observed in patient samples. Using fully annotated Affymetrix Gene 1.1 ST Array datasets (Northcott *et al*., 2012; Remke *et al*., 2011), we compared transcript levels of axon guidance genes in those Group 3 and 4 tumors that exhibit amplification or OE of *OTX2* and those that do not using a Pearson correlation coefficient and a FDR < 0.1. Forty axon guidance genes showed a significant correlation with *OTX2* expression (Table 1). Of these 40 genes, 27 (68%) were negatively correlated with *OTX2* expression (Table 1). Interestingly, SEMA signaling was also the most overrepresented pathway in this dataset with five genes (*SEMA6A*,* SEMA4D*,* NRP1*,* NRP2*, and *L1CAM*) all exhibiting a negative correlation with *OTX2* expression (Table 1). Cavalli *et al*. (2017) have recently shown that using a combination of genomewide DNA methylation and transcriptome profiling, Group 3 and Group 4 tumors could be further distinguished as separate entities. Using this larger dataset, we also evaluated survival based on expression of these 5 *SEMA* genes in Group 3 and Group 4 combined (*n* = 377), as well as Group 3 (*n* = 113) and Group 4 (*n* = 264) individually. Interestingly, univariable cox regression analysis of *SEMA4D* expression revealed a very significant inverse correlation with decreasing expression of *SEMA4D* in the combined Group 3 and Group 4 cohorts, and within Group 4 alone, but not in Group 3 alone (Table S6A). Similar results were obtained using top and bottom 20% *SEMA4D* expression within each subgroup, as lower levels of *SEMA4D* were associated with worse prognosis in Group 3 and Group 4 combined as well as Group 4 MB tumors alone (Fig. 3A). However, *SEMA6A*,* NRP1*,* NRP2*, and *L1CAM* were not significantly associated with outcome (Table S6B).

**Table 1:**
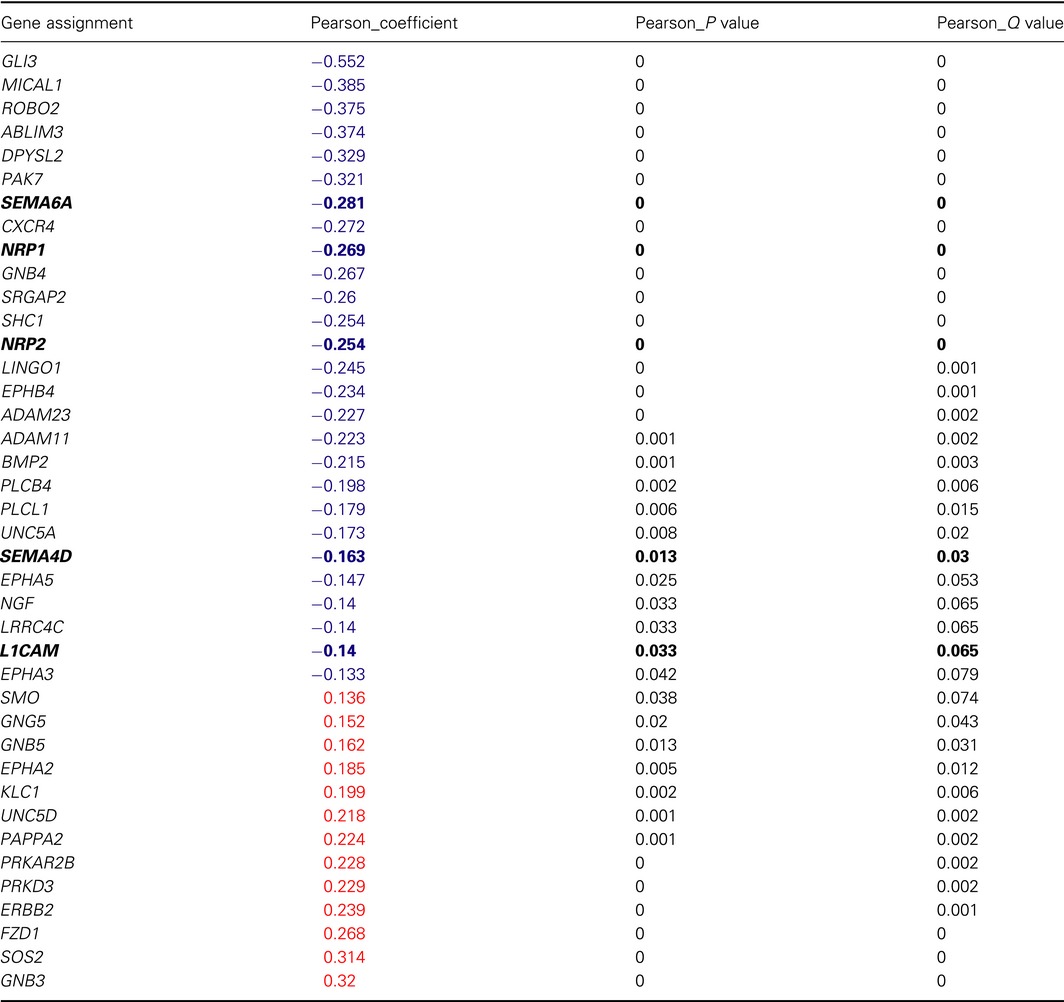
Correlation between OTX2 and expression of axon guidance genes in Group 3 and 4 MB patient samples. Genes exhibiting a statistically significant negative correlation are depicted in blue and those exhibiting a positive correlation are shown in red. FDR < 0.1

Semaphorin pathway genes are highlighted in bold.

Of the five SEMA pathway genes negatively correlated with OTX2 expression in patient samples, *L1CAM* and *NRP1* were most strongly upregulated following OTX2 KD in tumorspheres (Fig. S2) and *SEMA4D* was associated with prognosis (Fig. 3A). Thus, we chose these three genes for additional analyses at the protein level. Upon OTX2 KD, we observed an increase in L1CAM, NRP1, and SEMA4D protein concomitant with an increase in βIII‐tubulin by immunoblot in D283 and MB3W1 tumorspheres (Fig. 3B,C). Collectively, these results provide further support for the idea that axon guidance genes, particularly those belonging to the SEMA signaling pathway, are putative tumor suppressors in Group 3 and Group 4 MB and that SEMA4D is a novel prognostic indicator specifically in Group 4.

### 3.5 Increased SEMA gene levels are inversely correlated with tumorsphere formation, self‐renewal, and growth

**Figure 4:**
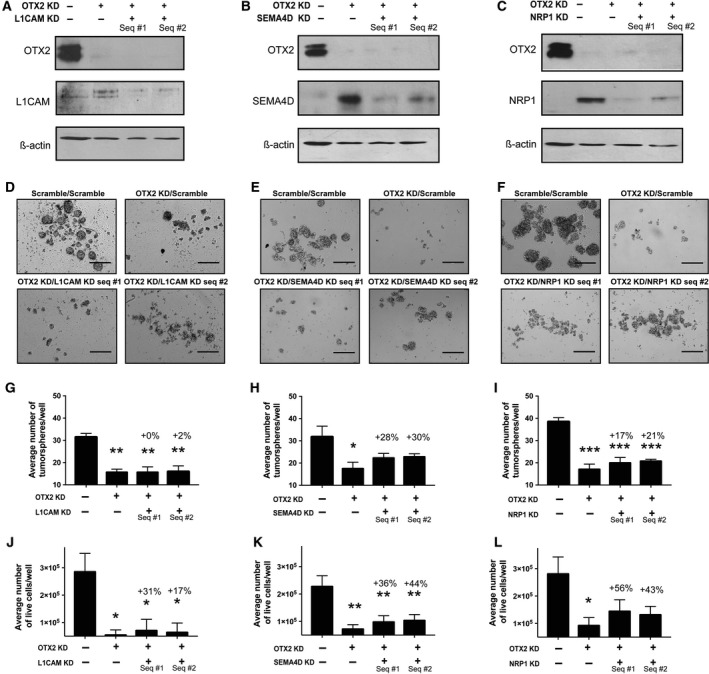
Decreased levels of semaphorin pathway genes in OTX2 KD cells result in a partial rescue of tumorsphere formation and growth. (A–C) Immunoblots depicting OTX2 and either L1CAM (A) SEMA4D (B) or NRP1 (C) protein levels following dual OTX2 and L1CAM, SEMA4D, or NRP1 knockdown in D283 tumorspheres over 5 days. β‐Actin serves as a loading control. (D–F) Representative images of D283 tumorspheres following dual OTX2 and either L1CAM (D), SEMA4D (E), or NRP1 (F) knockdown. Scale bar: 400 μm. (G–I) Quantification of tumorsphere number following dual OTX2 and either L1CAM (G), SEMA4D (H), or NRP1 (I) knockdown in D283 tumorspheres over 5 days. Note the partial recovery of tumorsphere number in the SEMA4D and NRP1 double knockdowns. Error bars: SEM. *P* < 0.05*, *P* < 0.01**, *P *< 0.001***. For all experiments, *N* = 3 or *N* = 4 biological replicates. (J–L) Quantification of total live cell number following dual OTX2 and either L1CAM (J), SEMA4D (K), or NRP1 (L) knockdown in D283 tumorspheres over 5 days. Error bars: SEM. *P* < 0.05*, *P *< 0.01**. For all experiments, *N* = 3–8 biological replicates.

Next, as proof of principle, we tested how modulation of OTX2 and SEMA pathway gene expression affects MB stem cell self‐renewal and growth *in vitro*. Recombinant human L1CAM, SEMA4D, and NRP1 Fc chimera proteins were added to D283 tumorsphere cultures (50–1000 ng·mL^−1^) and evaluated over passage (Fig. 3D–I). Increasing concentrations of recombinant SEMA4D Fc, L1CAM Fc, and NRP1 Fc chimera resulted in a dose‐dependent decrease in both primary and secondary tumorsphere formation (Fig. 3D–I) without affecting growth and viability (Fig. S4); however, only the highest concentration (1000 ng·mL^−1^) was significant. We also asked whether SEMA4D, NRP1, and L1CAM contribute to the OTX2 KD phenotype by performing dual OTX2/SEMA gene knockdown experiments in D283 tumorspheres (Fig. 4A–C). OTX2 was knocked down using 1 of our validated siRNA sequences at day 0 (s9931 or s9932, as these resulted in the most significant phenotypic change; Fig. 1), and then at day 2, cells were treated with either two independent SEMA4D, NRP1, or L1CAM siRNA sequences or a nonsilencing (scramble) siRNA negative control. While no rescue in tumorsphere formation or cell number was evident in OTX2 KD/L1CAM KD tumorspheres (Fig. 4D, G, J), we observed a partial rescue in both tumorsphere formation and cell number for both OTX2 KD/SEMA4D KD and OTX2 KD/NRP1 KD tumorspheres (Fig. 4E, F, H, I, K, L). Similar increases in tumorsphere size and cell number were observed following dual OTX2/SEMA4D or OTX2/NRP1 KD in MB3W1 tumorspheres (Fig. S5). For both D283 and MB3W1, there was no significant change in viability as measured by Trypan blue staining following dual KD (Fig. S6). Collectively, these results provide further evidence that SEMA genes are associated with a tumor‐suppressive role and contribute to the OTX2 KD phenotype in MB tumorspheres.

### 3.6 OTX2 limits RHO pathway activation

**Figure 5:**
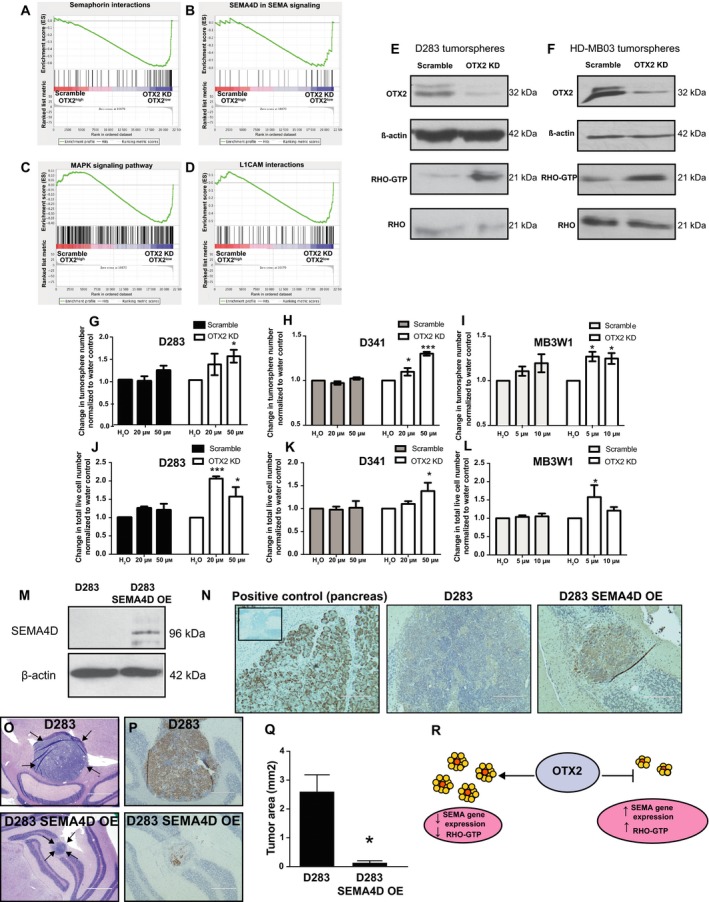
OTX2 levels are negatively correlated with activation of RHO signaling. (A–D) Gene set enrichment analysis (GSEA) demonstrating that genes associated with SEMA interactions (A), SEMA4D signaling (B), MAPK signaling (C), and L1CAM interactions (D) were enriched in genes sets that are downregulated in the scramble and upregulated in the OTX2 KD D283 tumorspheres. (E,F) Detection of the GTP‐bound form of active RHO in a pull‐down assay from scramble and OTX2 KD D283 (E) and HD‐MB03 (F) tumorsphere lysates. β‐Actin serves as a loading control for OTX2. Total RHO serves as a loading control for RHO‐GTP. (G–L) Treatment of D283 (G,J) and D341 (H,K) and MB3W1 (I,L) scramble (SC) and OTX2 KD tumorspheres with the RHO‐associated, coiled‐coil containing protein kinase (ROCK) inhibitor Y‐27632 results in a significant increase in tumorsphere number and total live cell number specifically in OTX2 KD cells relative to H_2_O controls, *N* = 3 biological replicates for D283 and MB3W1; *N* = 4 biological replicates for D341. Error bars: SEM. *P* < 0.05*, *P* < 0.001***. In addition to the treatment effects within the OTX2 KD cells, there was also a statistically significant difference between the scramble and the OTX2 KD tumorspheres in (H) (*P* < 0.001***) and (J) (*P* < 0.01**). Note that for MB3W1, higher concentrations were toxic; thus, the assays were performed at 5 and 10 μm. (M) Immunoblot validation of stable SEMA4D OE in D283 tumorspheres. β‐Actin serves as a loading control. (N) Representative images of SEMA4D staining in the pancreas (positive control for SEMA4D expression) (left) as well as tumors derived from D283 (middle) or D283 SEMA4D OE (right) following injection into NOD SCID mice. Inset: secondary antibody only negative control. Scale bar: 200 μm. (O) Representative images of tumors derived from D283 control or D283 SEMA4D OE tumorspheres following injection into the cerebellum of NOD SCID mice. Scale bar: 1000 μm. Arrows denote intracerebellar tumors from each. (P) Representative images of Ki67 staining in tumors derived from D283 control or D283 SEMA4D OE tumorspheres following injection into the cerebellum of NOD SCID mice. Scale bar: 400 μm. (Q) Quantification of tumor area in the vermis following intracerebellar injection of 2 × 10^5^ D283 control or D283 SEMA4D OE tumorsphere cells. Error bars: SEM. *P* < 0.05*. (R) Working model depicting the relationship between OTX2, SEMA gene expression, and RHO activity. OTX2 levels are inversely correlated with SEMA gene expression and RHO activity. Increased expression of SEMA genes and RHO activity is associated with decreased self‐renewal and growth and a more differentiated phenotype.

Next, we investigated the downstream pathways known to mediate the effects of SEMA and other axon guidance gene signaling such as RHO and MAPK (both ERK1/2 and p38) in OTX2 KD cells. Further interrogation of our gene expression profiling data revealed that RHO and MAPK signaling pathways were differentially expressed in OTX2^high^ relative to OTX2^low^ tumorspheres (Table S7). Genes associated with L1CAM interactions as well as SEMA interactions/SEMA4D signaling including the RHO pathway members *ROCK1*/*ROCK2* and several RHO guanine nucleotide exchange factors were significantly enriched in gene sets that were upregulated following OTX2 KD (Fig. 5A, B, D; Table S8). Similarly, genes associated with MAPK signaling (both ERK1/2 and p38) were enriched in gene sets that were upregulated in the OTX2 KD cells; however, there was also enrichment in the scramble controls (Fig. 5C). In support of these findings, we observed an increase in RHO activity in D283 tumorspheres as well as HD‐MB03 tumorspheres following OTX2 KD (Fig. 5E, F) while MAPK pathway activation (both ERK1/2 and p38) was inconsistent (data not shown).

To further interrogate the functional role of the RHO pathway in MB stem/progenitor cells, we treated scramble and OTX2 KD tumorspheres from D283, D341, and MB3W1 cells with the Y‐27632 ROCK inhibitor. While ROCK inhibition had no significant effect on tumorsphere formation or cell number in scramble controls, there was a statistically significant increase in OTX2 KD tumorsphere number and cell number in all three cell lines (Fig. 5G–L). Viability was significantly increased in D283 tumorspheres when treated with 20 μm ROCK only (data not shown). These results reveal a novel inhibitory role for the RHO pathway in MB stem/progenitor cells. Thus, both SEMA genes and their downstream effectors are negatively correlated with the OTX2‐driven phenotype in MB tumorspheres.

### 3.7 Stable overexpression of SEMA4D inhibits localized tumor growth *in vivo*

Finally, as SEMA4D is a novel prognostic indicator in Group 3 and Group 4 patients, is associated with a decrease in self‐renewal, and growth and is an upstream regulator of RHO activity, we evaluated the effect of this candidate gene on tumor growth *in vivo*. We generated stable D283 SEMA4D overexpressing (OE) tumorspheres (Fig. 5M) and injected 2 × 10^5^ D283 SEMA4D OE and corresponding D283 control tumorspheres into the cerebellum of NOD SCID mice (*N* = 4 for each). SEMA4D OE was sustained *in vivo* (Fig. 5N), and as expected, compared with D283 control tumors, D283 SEMA4D OE tumors displayed significantly less localized growth in the vermis as demonstrated by a decrease in Ki67 staining (Fig. 5O, P) as well as a significant decrease in tumor area (2.6 ± 0.6 mm^2^ in D283 vs. 0.1 ± 0.07 mm^2^ in D283 SEMA4D OE) (Fig. 5Q). However, two of the D283 SEMA4D OE animals exhibited extensive subarachnoid space tumor cell spread (data not shown), suggesting that SEMA4D may be contributing to a phenotypic switch *in vivo*.

## 4 Discussion

We have defined an OTX2‐driven stem cell program in Group 3 and Group 4 MB. Brain tumor cells grown in stem cell conditions more closely retain the genotype and phenotype of primary tumors compared with serum‐cultured lines (Lee *et al*., 2006). Interestingly, Di *et al*. (2005) previously showed that all *trans*‐retinoic acid (ATRA) downregulates OTX2 expression and inhibits OTX2+ MB cell growth *in vitro*. However, subsequent testing of ATRA and other retinoic acids in tumorspheres and in intracranial transplant models rendered the cells resistant to treatment (Bai *et al*., 2010). Thus, tumorspheres grown in stem cell‐enriched conditions are the most biologically relevant *in vitro* model system for characterizing the OTX2 regulatory network. This is supported by changes in OTX2 levels during the later stages of human cerebellar development where OTX2 is expressed in the more primitive progenitor cells of the external granular layer (EGL) but is not detected postnatally (de Haas *et al*., 2006).

We have identified novel associations between OTX2 and a large cohort of axon guidance genes and their downstream targets in Group 3 and Group 4 MB stem/progenitor cells. The majority of genes associated with axon guidance pathways were negatively correlated with OTX2 expression and self‐renewal suggesting a novel tumor‐suppressive role in these tumors. Importantly, this negative correlation was demonstrated in recently derived cell lines (Dietl *et al*., 2016; Milde *et al*., 2012) as well as primary patient samples with *SEMA4D* serving as a novel prognostic indicator in Group 4 MB tumors. The family of genes that regulate axon guidance has been found to play prominent roles in neuronal migration, motility, and tumor progression, but to our knowledge, it has never been associated with stem/progenitor cell populations in highly aggressive MB. Our results also extend current knowledge that axon guidance genes can undergo alternative splicing in MB tumors (Dubuc *et al*., 2012). Interestingly, several differentially expressed SEMA genes such as SEMA6A (Renaud *et al*., 2008), PLXNA2 (Renaud *et al*., 2008), NRP1 (Telley *et al*., 2016), and L1CAM (Huang *et al*., 2013) have been shown to play a role in cerebellar development further underscoring the notion that an early cerebellar stem/progenitor is the cell of origin for Group 3 and Group 4 MB. Indeed, a large number of neuronal differentiation genes (Table S3) including, but not limited to, *CBLN1*,* NEUROD1*,* NR2F1*, and *NRXN1* (Schuller *et al*., 2006; Uemura *et al*., 2010) have been implicated in cerebellar morphology and/or granule cell development thus further validating the biological relevance of the tumorsphere model system.

We propose a working model in which OTX2 promotes MB self‐renewal/growth and suppresses differentiation by inhibiting the expression of several classes of axon guidance genes, including SEMA pathway genes, and their downstream effectors (Fig. 5R). A recent study has shown that SEMA genes regulate brain tumor stem cell survival. Specifically, the SEMA ligand SEMA3C controls glioblastoma stem cell survival though RAC1 activation (Man *et al*., 2014), suggesting that the effect of SEMA genes on brain tumor stem cells is cell context specific. Moreover, as our dual OTX2/SEMA gene KD studies only resulted in a partial rescue of tumorsphere formation and cell growth, additional OTX2‐axon guidance signaling pathways will need to be explored. The partial rescue was not surprising given the fact that OTX2 KD resulted in expression changes in 3614 genes. For example, ephrin/EPH family genes were also negatively correlated with OTX2 expression in our global gene expression (Table S4) and patient sample datasets (Table 1). As EPH and SEMA signaling share many downstream targets including the RHO family of GTPases, the role of OTX2‐EPH signaling in regulating MB self‐renewal will need to be examined.

Our results demonstrate that OTX2 is either a direct or indirect repressor of SEMA gene expression. OTX2 binding at genomic regions of SEMA genes that lack OTX2 binding motifs suggests that OTX2 can cooperate with a complex of factors to restrict SEMA gene expression in Group 3 and Group 4 MB. While two SEMA genes exhibited OTX2 enrichment at TAATCT and/or TAATCC OTX2 binding motifs, these sequences are not specific to OTX2; thus, additional experiments to define the mechanism by which OTX2 binds to these axon guidance genes are needed. Interestingly, Bai *et al*. (2012) showed that OTX2 represses myogenic differentiation by binding to the 258 bp *MYOD1* core enhancer through its homeodomain in MB cells. In this study, the authors also suggested that OTX2 may recruit yet unidentified additional factors that will aid in suppressing multiple differentiation pathways (Bai *et al*., 2012). Bunt *et al*. (2012) proposed that the transcription factors NEUROD1 and NR2F1, both direct targets OTX2, may contribute to OTX2‐mediated regulation of differentiation, but the functional roles of these two proteins were not studied. Indeed, *NEUROD1* and *NR2F1* were both significantly upregulated following OTX2 KD in our MB tumorspheres (Table S3). In addition, OTX2 is associated with regulation of histone modifications (Bunt *et al*., 2013) and has recently been found to cooperatively control the active enhancer landscape in Group 3 MB (Boulay *et al*., 2017). Therefore, OTX2 may colocalize with specific histone modifications (i.e. H3K4me3 or H3K27me3) on select axon guidance pathway genes to regulate self‐renewal and differentiation. As axon guidance pathways have been shown to be critical regulators of invasion and metastasis in other cancers (Neufeld *et al*., 2016), it will also be imperative to evaluate the effects of SEMA pathway genes on Group 3 and Group 4 MB motility and metastasis. For example, the SEMA3B ligand has been shown to inhibit tumor cell growth while increasing metastatic dissemination in melanoma and nonsmall cell lung carcinoma cells (Rolny *et al*., 2008). OE of SEMA4D in our model system decreased localized tumor growth but an increase in subarachnoid space spread was observed. However, these studies were performed in a small number of animals and would need to be repeated with a larger cohort. Other axon guidance cues have shown variable effects on MB cell motility with NETRIN‐1 serving as a positive regulator of invasion (Akino *et al*., 2014), while SLIT2 inhibits MB invasion (Werbowetski‐Ogilvie *et al*., 2006). We have previously shown that higher self‐renewing MB tumorspheres exhibit a downregulated motility transcription program (Morrison *et al*., 2013). This raises the intriguing possibility that the effects of axon guidance genes on MB cells will be dependent on cellular phenotype.

Our results open up new avenues for studying the effects of the RHO family of small GTP‐binding proteins in MB stem/progenitor cells. SEMA4D signaling has been shown to either activate or inactivate RHO depending on the coupling of the PLXNB1 receptor with the ERBB2 or MET receptor tyrosine kinase (Swiercz *et al*., 2008). For example, through the involvement of a PLXN‐associated PDZ‐RHO‐GEF, RhoA activates cell migration via the PLXN‐ERBB2 complex in normal neural stem‐like cells (Swiercz *et al*., 2008). In our MB models, increased RHO activity is associated with a more differentiated phenotype, and this is consistent with previous findings in glioblastoma stem cells in which ROCK inhibitor treatment enhanced tumorsphere formation (Tilson *et al*., 2015).

In summary, we have found that OTX2 is a critical regulator of Group 3 and Group 4 self‐renewal and differentiation through modulation of a large cohort of axon guidance genes and downstream targets such as RHO activity. Our study offers novel mechanistic insights into MB self‐renewal and presents an informed framework to pursue novel targeted therapies aimed at axon guidance gene pathways to facilitate differentiation of MB cells.

## Author contributions

MS and TEW‐O contributed to conception and design, collection and/or assembly of data, data analysis and interpretation, manuscript writing, and final approval of manuscript. NT, LCM, and RK contributed to collection and/or assembly of data, and final approval of manuscript. MW and TM contributed to data collection and final approval of manuscript. JZ, GP, PS, VR, MRDB, and MDT contributed to data analysis and interpretation, and final approval of manuscript.

## Supporting information


**Fig. S1.** Knockdown of OTX2 in Group 3 and Group 4 MB decreases tumorsphere formation and self‐renewal.
**Fig. S2.** Semaphorin genes are negatively correlated with OTX2 expression in Group 3 and Group 4 MB cells.
**Fig. S3.** Axon guidance gene expression is upregulated following OTX2 knockdown in Group 3 and Group 4 MB cells.
**Fig. S4.** Recombinant semaphorin protein treatment does not significantly affect cell number or viability of D283 tumorspheres.
**Fig. S5.** Decreased levels of semaphorin pathway genes in MB3W1 OTX2 KD cells results in a partial rescue of tumorsphere formation and growth.
**Fig. S6.** Decreased levels of semaphorin pathway genes in D283 and MB3W1 OTX2 KD tumorspheres does not significantly affect viability.
**Table S1.** List of antibodies and concentrations used for Western blots.
**Table S2.** List of primers sequences used for qPCR.
**Table S3.** Neuronal differentiation genes that are significantly and differentially expressed following OTX2 knockdown in D283 tumorspheres.
**Table S4.** Axon guidance genes that are significantly and differentially expressed following OTX2 knockdown in D283 tumorspheres.
**Table S5.** Axon guidance pathway genes that were significantly and differentially expressed following OTX2 KD and the number of OTX2 binding peaks/overlaps within −5 kb to +2 kb of their transcriptional start sites.
**Table S6.** (A) Univariable cox regression analysis of survival by increasing *SEMA4D* expression across Group 3 and 4 medulloblastoma. (B) Statistical analyses of survival in patients exhibiting high *SEMA6A NRP1*,* NRP2*,* or L1CAM* gene expression relative to patients with low *SEMA6A*,* NRP1*,* NRP2 or L1CAM* gene expression.
**Table S7.** Gene Set Enrichment Analysis (GSEA) results for Reactome and KEGG databases identified pathways significantly enriched in gene sets that were downregulated in D283 Scramble relative to OTX2 KD tumorspheres.
**Table S8.** GSEA revealed that genes associated with SEMA4D signaling were enriched in gene sets that were downregulated in D283 scramble relative to OTX2 KD tumorspheres.
