# Supplementary material for: Characterization of a novel OTX2‐driven stem cell program in Group 3 and Group 4 medulloblastoma
Source: Mol Oncol. 2018 Mar 1;12(4):495–513. doi: 10.1002/1878-0261.12177 (PMC5891039; doi:10.1002/1878-0261.12177)
Supplement: Supplementary file 1 — Fig. S1. Knockdown of OTX2 in Group 3 and Group 4 MB decreases tumorsphere formation and self‐renewal. Fig. S2. Semaphorin genes are negatively correlated with OTX2 expression in Group 3 and Group 4 MB cells. Fig. S3. Axon guidance gene expression is upregulated following OTX2 knockdown in Group 3 and Group 4 MB cells. Fig. S4. Recombinant semaphorin protein treatment does not significantly affect cell number or viability of D283 tumorspheres. Fig. S5. Decreased levels of semaphorin pathway genes in MB3W1 OTX2 KD cells results in a partial rescue of tumorsphere formation and growth. Fig. S6. Decreased levels of semaphorin pathway genes in D283 and MB3W1 OTX2 KD tumorspheres does not significantly affect viability. Table S1. List of antibodies and concentrations used for Western blots. Table S2. List of primers sequences used for qPCR. Table S3. Neuronal differentiation genes that are significantly and differentially expressed following OTX2 knockdown in D283 tumorspheres. Table S4. Axon guidance genes that are significantly and differentially expressed following OTX2 knockdown in D283 tumorspheres. Table S5. Axon guidance pathway genes that were significantly and differentially expressed following OTX2 KD and the number of OTX2 binding peaks/overlaps within −5 kb to +2 kb of their transcriptional start sites. Table S6. (A) Univariable cox regression analysis of survival by increasing SEMA4D expression across Group 3 and 4 medulloblastoma. (B) Statistical analyses of survival in patients exhibiting high SEMA6A NRP1, NRP2, or L1CAM gene expression relative to patients with low SEMA6A, NRP1, NRP2 or L1CAM gene expression. Table S7. Gene Set Enrichment Analysis (GSEA) results for Reactome and KEGG databases identified pathways significantly enriched in gene sets that were downregulated in D283 Scramble relative to OTX2 KD tumorspheres. Table S8. GSEA revealed that genes associated with SEMA4D signaling were enriched in gene sets that were downregulated in D283 scramble [file MOL2-12-495-s001.pdf]

Characterization of a novel OTX2-driven stem cell program in Group 3 and Group 4 medulloblastoma

Margaret Stromecki, Nazanin Tatari, Ludivine Coudière Morrison, Ravinder Kaur, Jamie Zagozewski, Gareth Palidwor, Vijay Ramaswamy, Patryk Skowron, Matthias Wölfl, Till Milde, Marc R. Del Bigio, Michael D. Taylor, Tamra Werbowetski-Ogilvie

SUPPLEMENTARY FIGURE AND TABLE LEGENDS:

Supplementary Fig. 1: Knockdown of OTX2 in Group 3 and Group 4 MB decreases tumorsphere formation and self-renewal. (**A**). Western Blot validation of OTX2 knockdown in tumorspheres from the D341 MB cell line. (**B**). Representative images of tumorspheres at secondary passage following OTX2 knockdown in D341 cells. (**C-D**). Quantification of primary (**C**) and secondary (**D**) tumorsphere number in D341 tumorspheres following OTX2 knockdown. Error Bars: s.e.m. $p<0.05$*, $p<0.01$**, $p<0.001$***. (**E**). Western Blot validation of OTX2 knockdown in tumorspheres from MB3W1 tumorspheres. (**F**). Representative images of tumorspheres at secondary passage following OTX2 knockdown in MB3W1 cells. (**G-H**). Quantification of primary (**G**) and secondary (**H**) tumorsphere number in MB3W1 tumorspheres following OTX2 knockdown. Error Bars: s.e.m. $p<0.05$*, $p<0.001$***. For all experiments, N=3 biological replicates or independent transfections for each siRNA.

Supplementary Fig. 2: Semaphorin genes are negatively correlated with OTX2 expression in Group 3 and Group 4 MB cells. Expression of select semaphorin pathway genes (*SEMA4D, NRP1, L1CAM, SEMA6A, PLXNA2*) in D283 (**A**) D341 (**B**) and MB3W1 (**C**) MB tumorspheres following OTX2 knockdown using 3 siRNAs by qPCR. Error bars: s.e.m. N=3 biological replicates, n=9 technical replicates.

Supplementary Fig. 3: Axon guidance gene expression is upregulated following OTX2 knockdown in Group 3 and Group 4 MB cells. Expression of select axon guidance pathway genes (*SLIT2, EFNB2, EPHA3, EPHA5,* and *DCC*) in D283 (**A**) and D341 (**B**) tumorspheres following OTX2 knockdown using 3 siRNAs by qPCR. Error bars: s.e.m. N=3 biological replicates, n=9 technical replicates.

Supplementary Fig. 4: Recombinant semaphorin protein treatment does not significantly affect cell number or viability of D283 tumorspheres. (**A-C**). Total number of cells (upper) and viability (lower) in primary D283 tumorspheres following 5-day treatment with recombinant L1CAM Fc (**A**), SEMA4D (**B**) or NRP1 (**C**). (**D-F**) Total number of live cells (upper) and viability (lower) in secondary D283 tumorspheres following 5-day treatment with L1CAM Fc (**D**) SEMA4D (**E**) or NRP1 (**F**). For all experiments, N=4 biological replicates, n=20 L1CAM Fc, n=18 SEMA4D Fc and NRP1 Fc technical replicates. Error bars: s.e.m.

Supplementary Fig. 5: Decreased levels of semaphorin pathway genes in MB3W1 OTX2 KD cells results in a partial rescue of tumorsphere formation and growth. (**A**) Representative images of MB3W1 tumorspheres following dual OTX2 and either SEMA4D or NRP1 knockdown. Scale bar: 400 μm. (**B-C**). Quantification of tumorsphere number following dual OTX2 and either SEMA4D (**B**) or NRP1 (**C**) knockdown in MB3W1 tumorspheres over 5 days. Error Bars: s.e.m. $p<0.001$***. N=3 biological replicates, n=12 SEMA4D KD and NRP1 KD technical replicates. (**D-E**). Quantification of total live cell number following dual OTX2 and either SEMA4D (**D**) or NRP1 (**E**) knockdown in MB3W1 tumorspheres over 5 days. Error Bars: s.e.m. $p<0.05$*. N=4 biological replicates, n=8 SEMA4D KD and NRP1 KD technical replicates.

Supplementary Fig. 6: Decreased levels of semaphorin pathway genes in D283 and MB3W1 OTX2 KD tumorspheres does not significantly affect viability. (**A**). Viability in D283 tumorspheres following dual OTX2 and either SEMA4D (**left**) or NRP1 (**right**) knockdown over 5 days. Error Bars: s.e.m. N=7 and N=8 biological replicates for SEMA4D and NRP1 respectively. (**B**). Viability in MB3W1 tumorspheres following dual OTX2 and either SEMA4D (**left**) or NRP1 (**right**) knockdown over 5 days. Error Bars: s.e.m. N=4 biological replicates for SEMA4D and NRP1.

Supplementary Table 1: List of antibodies and concentrations used for Western blots

Supplementary Table 2: List of primers sequences used for qPCR

Supplementary Table 3: Neuronal differentiation genes that are significantly and differentially expressed following OTX2 knockdown in D283 tumorspheres. Transcripts differentially expressed at least 2-fold (up- or downregulated) and with a value of $P<0.05$ were considered significant

Supplementary Table 4: Axon guidance genes that are significantly and differentially expressed following OTX2 knockdown in D283 tumorspheres. Transcripts differentially expressed at least 2-fold (up- or downregulated) and with a value of $P<0.05$ were considered significant.

Supplementary Table 5: Axon guidance pathway genes that were significantly and differentially expressed following OTX2 KD and the number of OTX2 binding peaks /overlaps within -5kb-+2kb of their transcriptional start sites. Semaphorin genes significantly upregulated following OTX2 KD are highlighted in red.

Supplementary Table 6a: Univariable cox regression analysis of survival by increasing *SEMA4D* expression across Group 3 and 4 medulloblastoma.

Supplementary Table 6b: Statistical analyses of survival in patients exhibiting high *SEMA6A NRP1, NRP2, or L1CAM* gene expression relative to patients with low *SEMA6A, NRP1, NRP2 or L1CAM* gene expression. $P<0.05$ was considered significant.

Supplementary Table 7: Gene Set Enrichment Analysis (GSEA) results for Reactome and KEGG databases identified pathways significantly enriched in gene sets that were downregulated in D283 Scramble relative to OTX2 KD tumorspheres.

Supplementary Table 8: GSEA revealed that genes associated with SEMA4D signaling were enriched in gene sets that were downregulated in D283 scramble relative to OTX2 KD tumorspheres. All transcripts in the core enrichment were significantly different with a $P<0.05$.

Stromecki et al., Supplementary Figure 1

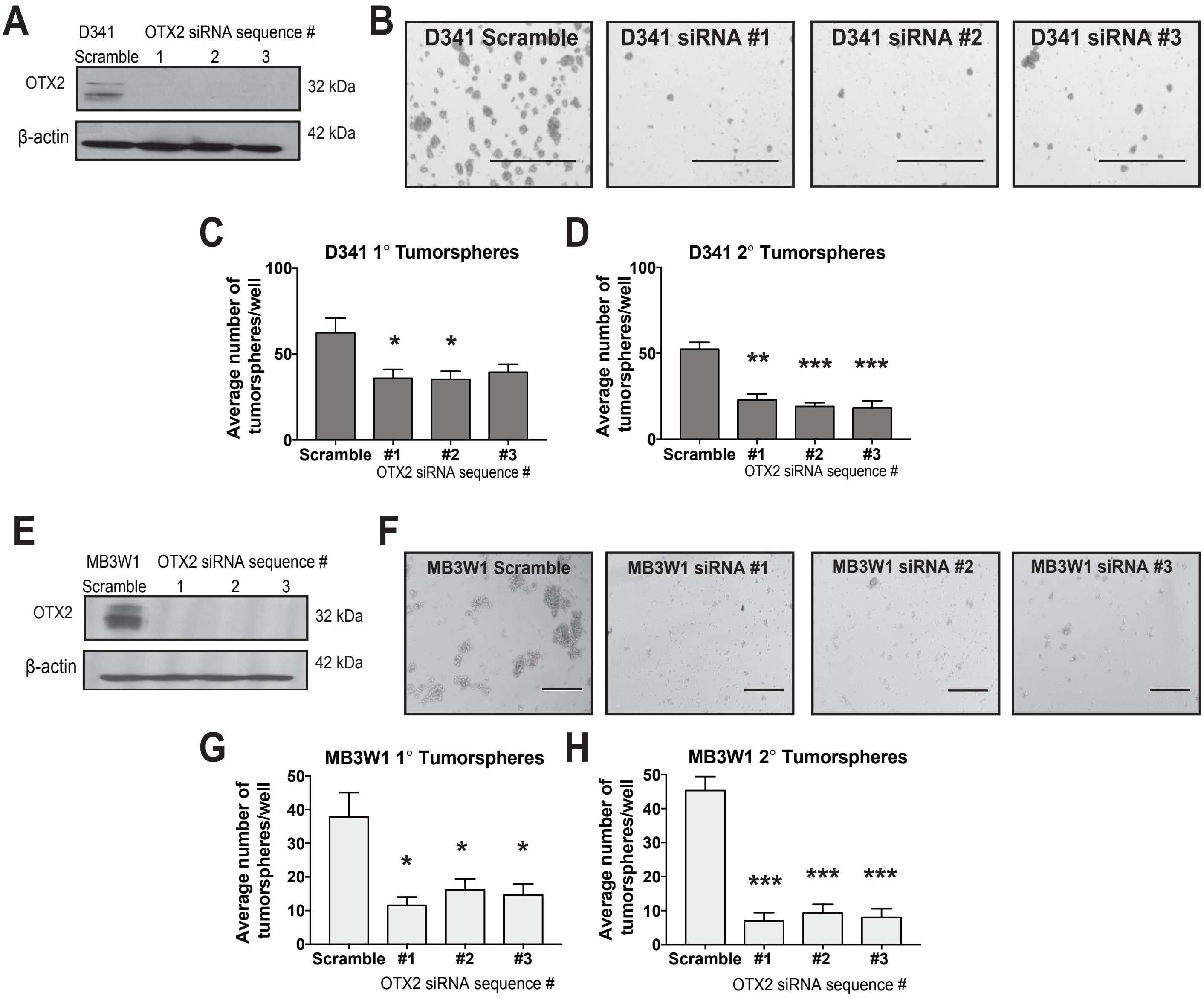

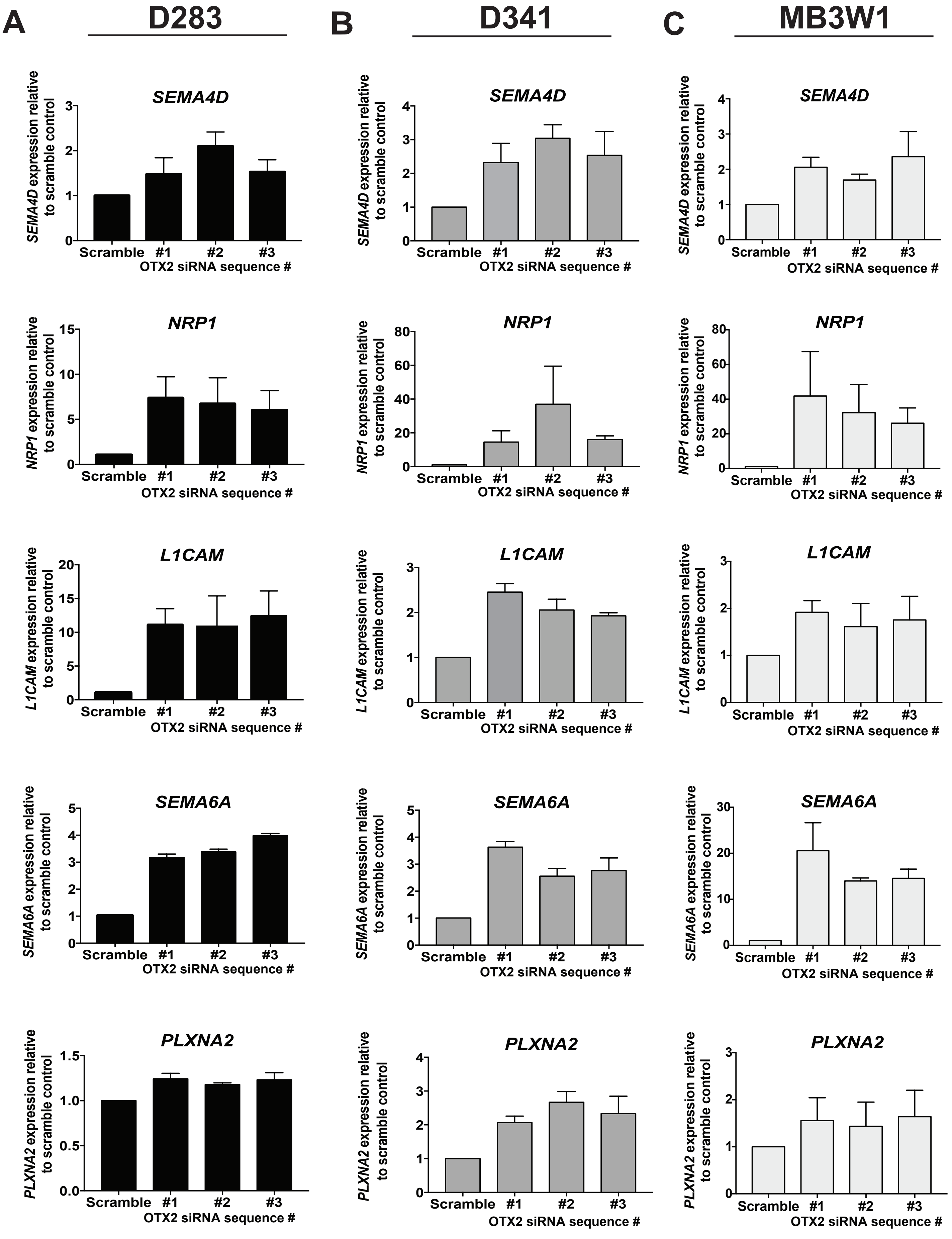
A
D283
B
D341
C
MB3W1
SEMA4D
SEMA4D expression relative to scramble control
NRP1
NRP1 expression relative to scramble control
L1CAM
L1CAM expression relative to scramble control
SEMA6A
SEMA6A expression relative to scramble control
PLXNA2
PLXNA2 expression relative to scramble control
Scramble
#1
#2
#3
OTX2 siRNA sequence #

Stromecki et al., Supplementary Figure 3

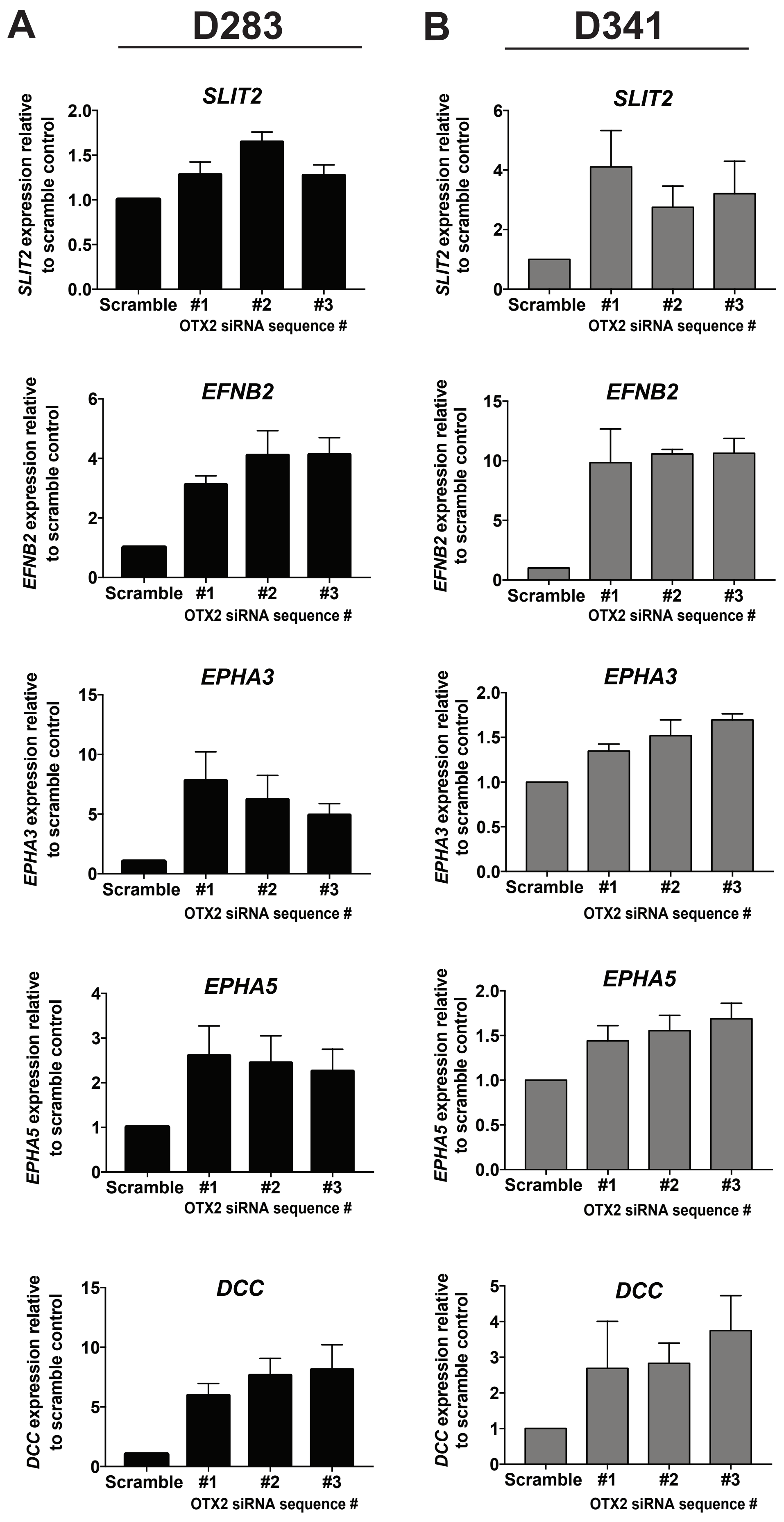

Stromecki et al., Supplementary Figure 4

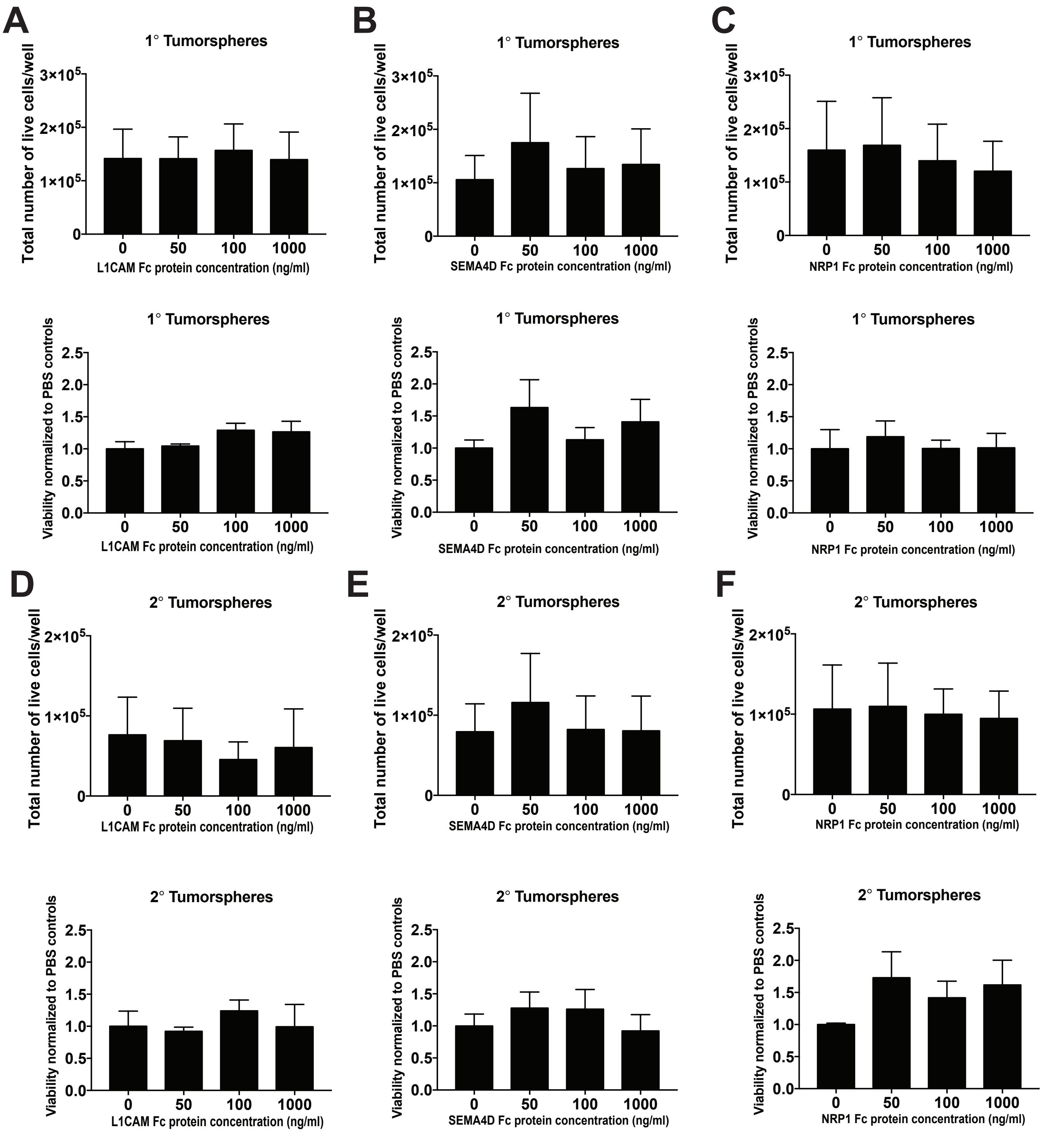

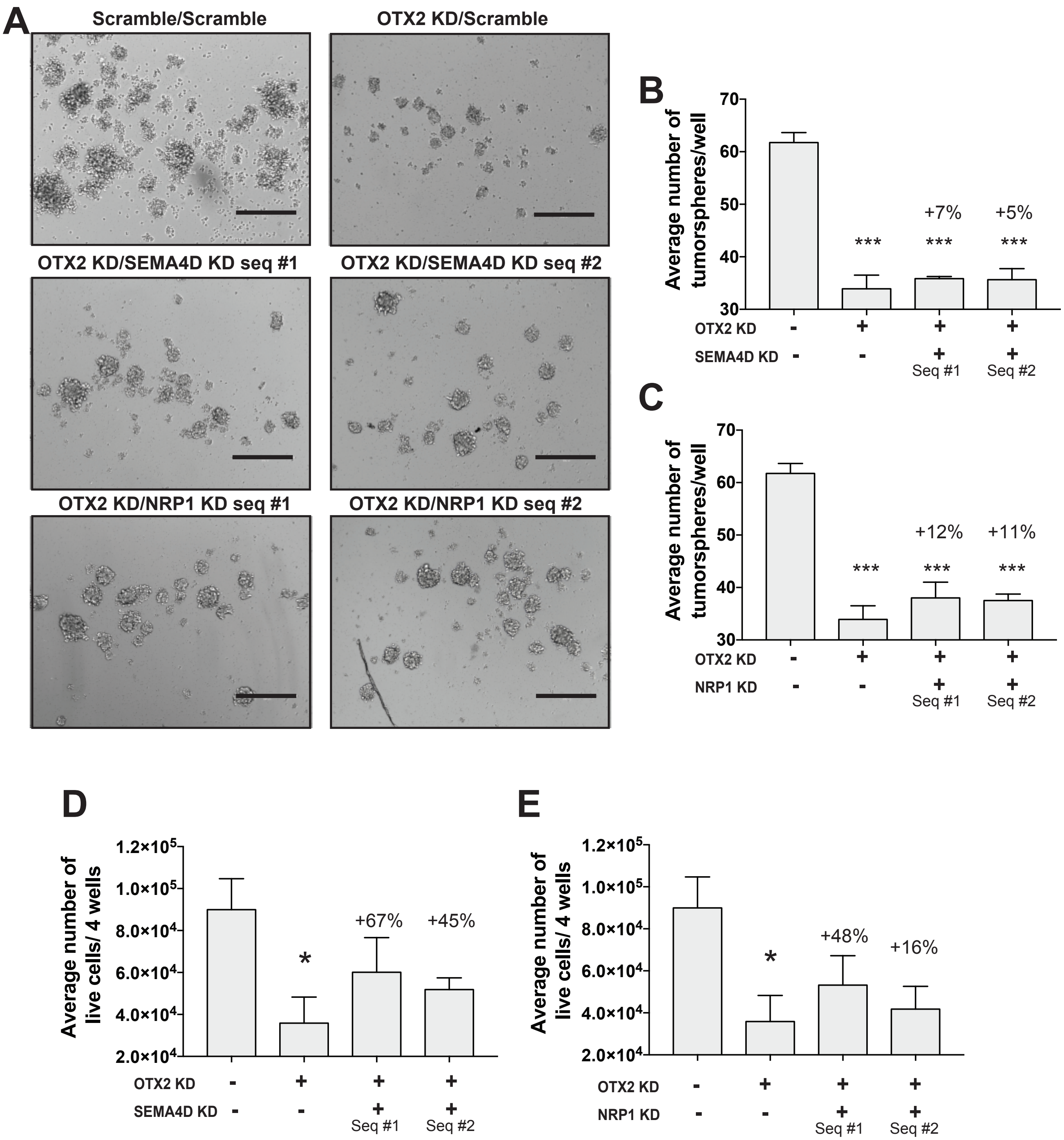
A
Scramble/Scramble
OTX2 KD/Scramble
OTX2 KD/SEMA4D KD seq #1
OTX2 KD/SEMA4D KD seq #2
OTX2 KD/NRP1 KD seq #1
OTX2 KD/NRP1 KD seq #2
B
Average number of tumorspheres/well
70
60
50
40
30
***
+7%
***
+5%
***
OTX2 KD - + + +
SEMA4D KD - - + +
Seq #1
Seq #2
C
Average number of tumorspheres/well
70
60
50
40
30
***
+12%
***
+11%
***
OTX2 KD - + + +
NRP1 KD - - + +
Seq #1
Seq #2
D
Average number of live cells/ 4 wells
1.2×10^5
1.0×10^5
8.0×10^4
6.0×10^4
4.0×10^4
2.0×10^4
*
+67%
+45%
OTX2 KD - + + +
SEMA4D KD - - + +
Seq #1
Seq #2
E
Average number of live cells/ 4 wells
1.2×10^5
1.0×10^5
8.0×10^4
6.0×10^4
4.0×10^4
2.0×10^4
*
+48%
+16%
OTX2 KD - + + +
NRP1 KD - - + +
Seq #1
Seq #2

Stromecki et al., Supplementary Figure 6

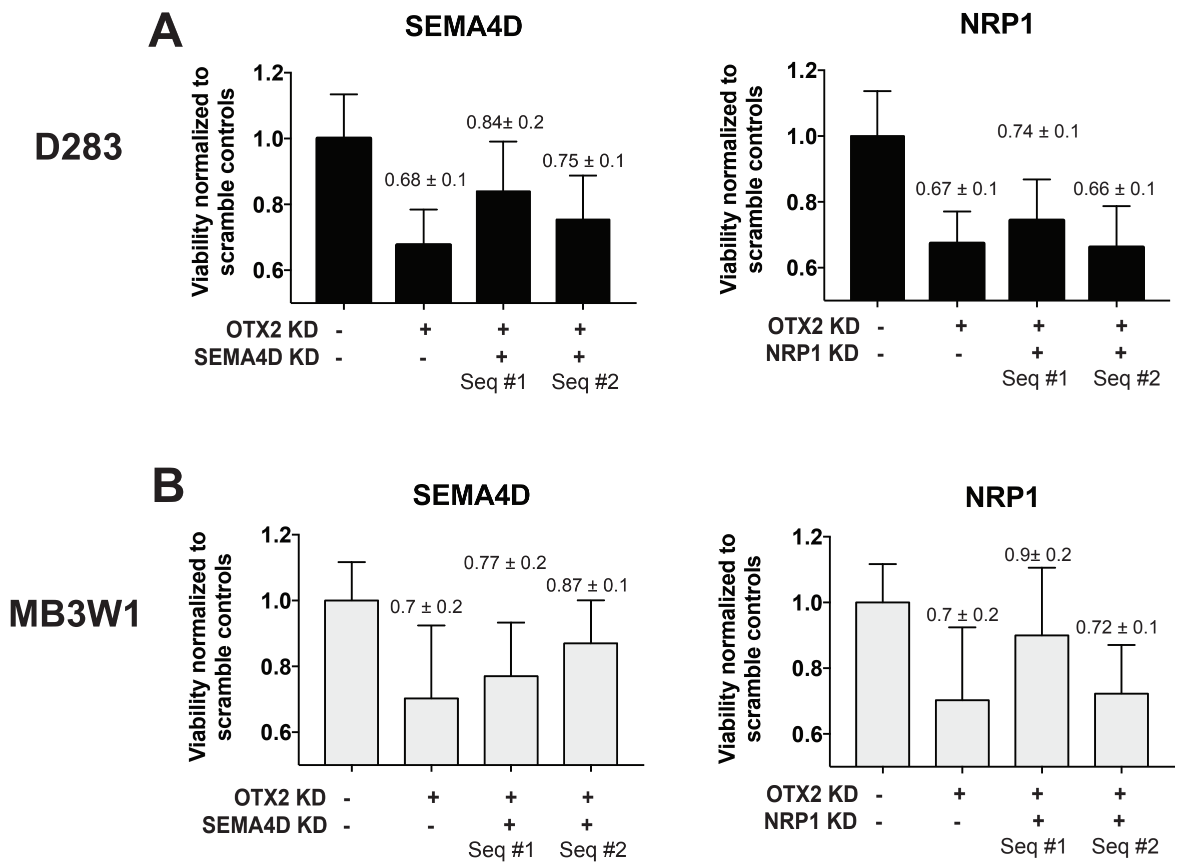

Supplementary Table 1: List of antibodies and concentrations used for Western blots

Gene	Concentration	Source and catalogue number
**β-actin**	1:1000	Abcam Inc., (ab8226)
**SEMA4D**	1:500	Abcam Inc., (ab134128)
**L1CAM**	1:500	Gene Tex, (GTX23200)
**NRP1**	1:500	Abcam Inc., (ab81321)
**ßIII Tubulin**	1:1000	R&D Systems, (MAB1195)
**OTX2**	1:500	Abcam Inc., (ab21990)
**RHO**	1:500	Thermo Fisher, (1862332)
**Peroxidase AffiniPure Donkey Anti-Rabbit IgG (H+L)**	1:5000	Jackson ImmunoResearch Laboratories Inc., (711-035-152)
**Goat Anti-Mouse IgG H&L (HRP)**	1:5000	Abcam Inc., (ab6789)

Supplementary Table 2: List of primer sequences used for qPCR

Gene	Forward sequence	Reverse sequence
GAPDH	3’- GAAATCCCATCACCATCTTCCAGG -5’	3’-GCAAATGAGCCCCAGCCT TCTC -5’
OTX2	5’-GAGGTGGCACTGAAAATCAAC-3’	5’-TCTTCTTTTTGGCAGGTCTCA-3’
TUJ1	5’-GGCCTTTGGACATCTCTTCA-3’	5’-TCGCAGTTTTCACACTCCTTC-3’
MAP2	5’-ATTCTGGCAGCAGTTCTCAAA-3’	5’-TGCTTCCTCGGTTAGAGACAA-3’
SEMA4D	5’-CGTCATGGTTGATGGAGAACT-3’	5’- AGCCAAGGGATTGCATATTCT-3’
L1CAM	5’-AAATGGCTGTGAAGACCAATG-3’	5’-GATGAAGCAGAGGATGAGCAG-3’
NRP1	5’-CGATTTGGAGGACAGAGACTG-3’	5’-GGGGCTATCTTTCCACAGAAC-3’
SEMA6A	5’-TTTGTTCAAGCCGTGGATTAC-3’	5’-GCCACTCTTGGGAAAACTACC-3’
PLXNA2	5’-ACCATCACACAGGTCAAGGAG-3’	5’-CACTCCAAGTCCATGTCCACT-3’
SLIT2	5’-TGAATTTACCGTGTTGGAAGC-3’	5’-CAGATGCTCCTTCAAATGCTC-3’
DCC	5’-TACTGGACCACCTTCCAACTG-3’	5’-CCTCACATGAAGAGAGCTTGG-3’
EPHA3	5’-GGAAGAGATCAGTGGTGTGGA-3’	5’-TTTTGACTGTGGTCCATGACA-3’
EPHA5	5’-ACAAAGGAAGCCAAATCACCT-3	5’-GGTAGAAACCCAAAGGCAGAC-3’
EPHB2	5’-GACTCCACTACAGAGACTGCT-3’	5’-TCTCATCGTAGCCACTCACCT-3’

Supplementary Table 3: Neuronal differentiation genes that are significantly and differentially expressed following OTX2 knockdown in D283 tumorspheres

Gene Assignment	Predictive effect on neuronal differentiation (based on measurement direction and literature compiled in Ingenuity® Knowledge Base)	Fold change
DPYSL2	Increased	2.284692494
NEUROD2	Increased	2.470837274
NTRK3	Increased	3.721798631
HMGB2	Increased	0.334250124
EFNA3	Increased	3.294364069
GAP43	Increased	10.4468783
MID1	Increased	0.398320048
NRXN1	Increased	7.459091891
NSG1	Increased	2.187070915
CNR1	Increased	9.787904329
EPHA4	Increased	2.713208655
LRP1	Increased	2.435132037
NR2F1	Increased	3.140512475
KLF7	Increased	2.797232165
DLG5	Increased	2.100889088
ACTL6B	Increased	2.239225777
CNTNAP2	Increased	9.493894381
NCAM2	Increased	13.78473856
TSC1	Increased	2.037782393
PAX6	Increased	3.031433133
RELN	Increased	4.00277355
SEMA6A	Increased	5.544279543
NUMBL	Increased	2.0265138
SDK1	Increased	2.396618043
CRMP1	Increased	8.190775203
DPYSL3	Increased	2.261061134
SNCA	Increased	5.95045505
DPYSL5	Increased	2.223758315
CIT	Increased	0.377356492
KIDINS220	Increased	2.979354926
SLIT2	Increased	3.365917929
KIF3A	Increased	2.844154821
GRIA1	Increased	6.520578659
BCL2	Increased	2.63170905
FNBP1	Increased	2.488023307
KIF1A	Increased	3.38932974
PDGFRA	Increased	4.982949662
RIMS1	Increased	4.161201066
APP	Increased	2.360348687
KALRN	Increased	4.806544198
ELAVL4	Increased	26.6857934
FYN	Increased	3.280691645

mir-181	Increased	2.987626914
CAMK4	Increased	3.431882122
TRIP10	Increased	0.219912269
CAMK1G	Increased	3.020945171
NEUROD1	Increased	2.193143177
CDK5R1	Increased	2.756810306
ADGRB2	Increased	2.494931144
MAPT	Increased	4.17564771
NEUROG2	Increased	2.797232165
SEMA4D	Increased	3.45575275
HES1	Increased	0.400257405
ID1	Increased	0.4181232
NEFH	Increased	0.291587342
RYR2	Increased	11.03488737
TNFRSF21	Increased	0.494142826
PRKCE	Increased	3.396384986
TBR1	Increased	2.698205069
CHRNA1	Increased	0.410940094
NEUROD6	Increased	2.946495372
SV2A	Increased	2.352182501
MAP6	Increased	12.92417244
ADGRL3	Increased	10.70342044
PCYT1B	Increased	2.200757219
LRRTM2	Increased	3.415270858
ASCL1	Increased	3.640155296
ADGRB3	Increased	2.040609318
CLSTN2	Increased	6.315951094
SOX11	Increased	2.380063393
NLGN3	Increased	2.136130816
DAGLA	Increased	2.319799309
SLITRK5	Increased	2.265767771
CAPRIN2	Increased	2.926142441
TENM4	Increased	8.24774655
LRRN3	Increased	6.050271989
FLRT1	Increased	4.23393758
CLSTN3	Increased	2.233025924
LRRTM1	Increased	4.334903867
CBLN2	Increased	2.11696879
NEUROD4	Increased	4.401514437
RIMS3	Increased	2.713208655
EPHB2	Increased	2.72829567
SEPT4	Decreased	0.276241121
ERBB2	Decreased	0.438910899
SULF1	Decreased	0.458819941
MUSK	Decreased	0.368311921
BMP7	Decreased	0.414659773
EEF2K	Decreased	0.281654807
PPP3CA	Decreased	2.488023307
RIT1	Decreased	2.051956291
DOCK10	Decreased	0.403880389

SMAD3	Decreased	0.44844408
TGFB1	Decreased	0.279709275
FGF2	Decreased	0.281069731
FGFR2	Decreased	0.248445273
SDC2	Decreased	0.492433221
COL25A1	Decreased	0.5
GDI1	Decreased	2.09216988
KIF23	Decreased	0.146807746
DCX	Decreased	5.241573615
SLC12A2	Decreased	0.334713814
SERPINF1	Decreased	0.195467411
SYNGAP1	Decreased	2.522754818
RTN4	Decreased	2.057653416
CBLN1	Decreased	0.342695701
GAS7	Decreased	0.185436867
RAPGEF2	Decreased	3.655325801
NTF3	Decreased	0.321078952
DCLK1	Decreased	4
RYR1	Decreased	0.483973513
CHRNA7	Decreased	0.295452887
GLI3	Decreased	0.243163737
KLF9	Decreased	0.219912269
NGF	Decreased	0.220064753
GEM	Decreased	0.268501118
L1CAM	Decreased	5.105315075
MYO5A	Decreased	2.324628215
IGF1	Decreased	0.467163673
DOCK1	Decreased	0.464902471
MAPK8IP1	Decreased	2.67585511
PARD3	Decreased	0.431071773
PLPPR5	Decreased	0.336108749
RAB17	Decreased	0.16747295
REM2	Decreased	2.270484204
ROBO2	Decreased	2.406606052
MDGA1	Decreased	2.236123702
BARHL2	Affected	2.202283196
LRP4	Affected	0.400812665
NYAP2	Affected	9.553305497
CELSR2	Affected	4.597979392
CPT1C	Affected	2.236123702
PCDHB9	Affected	2.020902893
RNF165	Affected	3.073750363
PRICKLE2	Affected	0.267757706
BRSK1	Affected	2.043440165
PCDHB14	Affected	3.673104649
ATL1	Affected	2.37676621
PDZRN3	Affected	0.321747312
GPM6A	Affected	2.430073584
LRRC7	Affected	22.7059737
RUFY3	Affected	2.496661098

SHTN1	Affected	4.717426369
NSMF	Affected	4.319906239
CCDC88A	Affected	2.069095163
BBS4	Affected	0.395842933
CLMN	Affected	0.221288441
FEZF2	Affected	2.687006851
SEZ6	Affected	2.944453724
TULP1	Affected	0.385552706
PCDH8	Affected	2.360348687
CACNA1F	Affected	0.406126198
CNTN4	Affected	5.158673003
CPEB4	Affected	2.150988781
ATCAY	Affected	11.08855909
PREX2	Affected	0.441351498
PLXND1	Affected	2.284692494
LRRC4C	Affected	3.69609029
CASP6	Affected	0.480630464
GABRA5	Affected	0.252437824
GFRA1	Affected	2.615342697
SPR	Affected	0.272815969
CACNA2D2	Affected	8.299359493
HSPB1	Affected	0.407253782
SYN1	Affected	2.599078125
LZTS1	Affected	2.350552657
MAP1B	Affected	2.711328654
NPTXR	Affected	22.54913208
SLC1A3	Affected	0.257563488
NCAN	Affected	4.422922613
SHC1	Affected	0.486327474
NOTCH1	Affected	0.384751805
PARD6B	Affected	0.283417353
ETV4	Affected	0.483973513
SYP	Affected	2.125791349
DMD	Affected	0.498615626
LAMA1	Affected	0.242322454
SNAP91	Affected	4.948529915
CACNB4	Affected	0.489370825
SNCB	Affected	4.319906239
MPP5	Affected	0.340328529
CD44	Affected	2.615342697
GJA1	Affected	0.095127086
BTG3	Affected	0.334250124
HCN1	Affected	3.048289661
NRG1	Affected	3.60500185
PCDHB13	Affected	4.456774603
CXCR4	Affected	0.35013908
EPB41L3	Affected	3.977880675
DNER	Affected	4.707626949
NR4A2	Affected	3.906834116
CTNNA2	Affected	2.327853069

CAST	Affected	0.41065535
TP73	Affected	5.045509635
SPTBN4	Affected	2.507066041
NRP2	Affected	5.392670927
PLXNA3	Affected	3.431882122
ONECUT1	Affected	4.352969767
THRA	Affected	3.928558381
RAB3A	Affected	2.353813474
ERBB3	Affected	0.416099367
ARHGEF28	Affected	2.170458744
MYOD1	Affected	0.172898829
LOX	Affected	0.35404386
RDX	Affected	0.498615626
CADM1	Affected	2.096524951
C3	Affected	0.190650207
UNC5A	Affected	3.647732662
UGT8	Affected	0.335875856
FZD5	Affected	0.456599125
VIM	Affected	0.355765865
OPTN	Affected	3.518596304
WNT7B	Affected	3.035638506
VLDLR	Affected	0.280097304
DISC1	Affected	0.278548413
COL4A1	Affected	0.240148716
PLPPR4	Affected	0.409802304
DCC	Affected	19.91798777
NRP1	Affected	4.525257851
GABRG2	Affected	5.172995728
ARHGAP33	Affected	3.786854525
CLU	Affected	0.393926943
CACNA1S	Affected	0.47204621
RAB29	Affected	0.206183387
MAP2	Affected	17.31559431
TOP2B	Affected	2.046274939
PRDM1	Affected	0.285982743
CDC20	Affected	0.178376813
LIF	Affected	0.414659773
SRCIN1	Affected	2.633533844
SRGAP2	Affected	2.106722072
SIPA1L1	Affected	2.049113646
HERC1	Affected	2.724516069
PDLIM5	Affected	0.454074209
EFNB2	Affected	8.456144324
WEE1	Affected	0.340800652
SIX4	Affected	0.346517471
BMP2	Affected	0.166200889
CACNA1A	Affected	14.89751712
LYN	Affected	0.185436867
PITPNA	Affected	0.47237352
NR2F6	Affected	0.469435874

NR1D1	Affected	2.30697121
KIF20B	Affected	0.33985706
PAK3	Affected	5.540437872
FNBP1L	Affected	2.648177821
PCDHB8	Affected	2.049113646
PCDHB10	Affected	4.23393758
PCDHB11	Affected	2.873880353
EPHA3	Affected	35.97692556
LAMB2	Affected	0.404160434
LINGO1	Affected	2.153972752

**Note: 85 of 252 differentially expressed genes have measurement direction consistent with an increase in neuronal development or differentiation, Z-score: 3.2

Supplementary Table 4: Axon guidance genes that are significantly and differentially expressed following OTX2 knockdown in D283 tumorspheres

Gene Assignment	Entrez Gene Name	Fold change
ABLIM3	actin binding LIM protein family, member 3	0.358488812
ADAM11	ADAM metallopeptidase domain 11	4.629960868
ADAM23	ADAM metallopeptidase domain 23	2.298989696
ADAMTS7	ADAM metallopeptidase with thrombospondin type 1 motif, 7	2.484576564
ARPC1B	actin related protein 2/3 complex, subunit 1B, 41kDa	0.467163673
BMP1	bone morphogenetic protein 1	2.38998241
BMP2	bone morphogenetic protein 2	0.166200889
BMP7	bone morphogenetic protein 7	0.414659773
C9orf3	chromosome 9 open reading frame 3	0.428390977
CXCR4	chemokine (C-X-C motif) receptor 4	0.35013908
DCC	DCC netrin 1 receptor	19.91798777
DOCK1	dedicator of cytokinesis 1	0.464902471
DPYSL2	dihydropyrimidinase-like 2	2.284692494
DPYSL5	dihydropyrimidinase-like 5	2.223758315
EFNA3	ephrin-A3	3.294364069
EFNA4	ephrin-A4	0.178624267
EFNB2	ephrin-B2	8.456144324
EPHA2	EPH receptor A2	0.18595172
EPHA3	EPH receptor A3	35.97692556
EPHA4	EPH receptor A4	2.713208655
EPHA5	EPH receptor A5	29.79503423
EPHB2	EPH receptor B2	2.72829567
EPHB4	EPH receptor B4	0.273763118
ERBB2	erb-b2 receptor tyrosine kinase 2	0.438910899
FYN	FYN proto-oncogene, Src family tyrosine kinase	3.280691645
FZD1	frizzled class receptor 1	0.411510173
FZD5	frizzled class receptor 5	0.456599125
FZD7	frizzled class receptor 7	0.08207007
GLI3	GLI family zinc finger 3	0.243163737
GNAO1	guanine nucleotide binding protein (G protein), alpha activating activity polypeptide O	5.747760706
GNB3	guanine nucleotide binding protein (G protein), beta polypeptide 3	0.257206677
GNB4	guanine nucleotide binding protein (G protein), beta polypeptide 4	0.294226684
GNB5	guanine nucleotide binding protein (G protein), beta 5	2.131693472
GNG2	guanine nucleotide binding protein (G protein), gamma 2	3.020945171
GNG3	guanine nucleotide binding protein (G protein), gamma 3	6.143241079
GNG5	guanine nucleotide binding protein (G protein), gamma 5	0.40584479
GNG11	guanine nucleotide binding protein (G protein), gamma 11	0.367801686
GNG12	guanine nucleotide binding protein (G protein), gamma 12	0.300200857
IGF1	insulin-like growth factor 1 (somatomedin C)	0.467163673
ITGA4	integrin, alpha 4 (antigen CD49D, alpha 4 subunit of VLA-4 receptor)	2.25792881
KALRN	kalirin, RhoGEF kinase	4.806544198
KLC1	kinesin light chain 1	2.164449289

L1CAM	L1 cell adhesion molecule	5.105315075
LINGO1	leucine rich repeat and Ig domain containing 1	2.153972752
LRRC4C	leucine rich repeat containing 4C	3.69609029
MICAL1	microtubule associated monooxygenase, calponin and LIM domain containing 1	2.425025638
MYL4	myosin, light chain 4, alkali; atrial, embryonic	0.357000995
NGF	nerve growth factor (beta polypeptide)	0.220064753
NRP1	neuropilin 1	4.525257851
NRP2	neuropilin 2	5.392670927
NTF3	neurotrophin 3	0.321078952
NTRK3	neurotrophic tyrosine kinase, receptor, type 3	3.721798631
PAK3	p21 protein (Cdc42/Rac)-activated kinase 3	5.540437872
PAK7	p21 protein (Cdc42/Rac)-activated kinase 7	4.688089135
PAPPA2	pappalysin 2	0.147624083
PLCB4	phospholipase C, beta 4	9.428315262
PLCD3	phospholipase C, delta 3	0.392292049
PLCD4	phospholipase C, delta 4	3.267075964
PLCE1	phospholipase C, epsilon 1	0.341036959
PLCG1	phospholipase C, gamma 1	2.403272099
PLCL1	phospholipase C-like 1	4.500233939
PLXNA2	plexin A2	3.43664302
PLXNA3	plexin A3	3.431882122
PLXND1	plexin D1	2.284692494
PPP3CA	protein phosphatase 3, catalytic subunit, alpha isozyme	2.488023307
PRKAR2B	protein kinase, cAMP-dependent, regulatory, type II, beta	2.486299338
PRKCE	protein kinase C, epsilon	3.396384986
PRKD3	protein kinase D3	0.459456442
PTCH2	patched 2	3.164549205
ROBO2	roundabout guidance receptor 2	2.406606052
RRAS	related RAS viral (r-ras) oncogene homolog	0.323985241
RTN4	reticulon 4	2.057653416
SDC2	syndecan 2	0.492433221
SEMA4D	sema domain, immunoglobulin domain (Ig), transmembrane domain (TM) and short cytoplasmic domain, (semaphorin) 4D	3.45575275
SEMA6A	sema domain, transmembrane domain (TM), and cytoplasmic domain, (semaphorin) 6A	5.544279543
SEMA6C	sema domain, transmembrane domain (TM), and cytoplasmic domain, (semaphorin) 6C	2.8108374
SHC1	SHC (Src homology 2 domain containing) transforming protein 1	0.486327474
SLIT1	slit guidance ligand 1	13.04115732
SLIT2	slit guidance ligand 2	3.365917929
SMO	smoothened, frizzled class receptor	0.422786144
SOS2	son of sevenless homolog 2 (Drosophila)	2.033549347
SRGAP2	SLIT-ROBO Rho GTPase activating protein 2	2.106722072
TUBB6	tubulin, beta 6 class V	0.284204243
TUBB2A	tubulin, beta 2A class IIa	2.358713185
TUBB2B	tubulin, beta 2B class IIb	2.00416321
TUBB4A	tubulin, beta 4A class IVa	2.124318373
UNC5A	unc-5 netrin receptor A	3.647732662
UNC5B	unc-5 netrin receptor B	2.599078125
UNC5D	unc-5 netrin receptor D	8.094825608
WNT7B	wingless-type MMTV integration site family, member 7B	3.035638506

Supplementary Table 5: Axon guidance pathway genes that were significantly and differentially expressed following OTX2 KD and the number of OTX2 binding peaks /overlaps within -5kb-+2kb of their transcriptional start sites

Gene Assignment	Fold change	Overlaps/OTX2-binding peaks
UNC5B	2.599856508	5
SLIT2	3.365945926	4
EFNB2	8.458995374	3
SEMA6A	5.542465942	3
PLXNA2	3.436757362	3
LRRC4C	3.696844429	3
KLC1	2.163857258	3
IGF1	0.467094383	3
SHC1	0.486217368	3
FZD5	0.456667281	3
GNG12	0.30012554	3
GNG3	6.14369672	2
NRP1	4.525936467	2
SEMA6C	2.811277756	2
FZD1	0.411479558	2
EPHA4	2.713058833	2
PLCE1	0.340932018	2
PLCD3	0.392306551	2
SRGAP2	2.107118816	2
NRP2	5.392071649	2
PLCL1	4.499103846	2
RRAS	0.324050897	2
GNG11	0.367793953	2
EPHA5	29.7964111	1
DCC	19.9143847	1
FZD7	0.082085886	1
EPHA3	35.96546453	1
UNC5D	8.093918561	1
PLCB4	9.425849634	1
SLIT1	13.04052457	1
GNB3	0.257122066	1
KALRN	4.807760401	1
EPHA2	0.185952064	1
BMP2	0.166236374	1
CXCR4	0.350026972	1

GNB4	0.294242592	1
EFNA3	3.293833584	1
PPP3CA	2.488226239	1
NGF	0.220082042	1
PTCH2	3.165072031	1
UNC5A	3.648270412	1
MYL4	0.356958518	1
WNT7B	3.035941518	1
DPYSL5	2.223862104	1
TUBB6	0.284219872	1
EFNA4	0.178674171	1
PLCD4	3.266598176	1
SDC2	0.492339251	1
SOS2	2.033516927	1
BMP7	0.414541181	1
MICAL1	2.42543301	1
GNG5	0.405713159	1
PRKD3	0.45951462	1
GNB5	2.131834339	1
ERBB2	0.438769353	1
ARPC1B	0.46707323	1
L1CAM	5.106598619	0
PAPPA2	0.14758633	0
GNAO1	5.746351854	0
PLXNA3	3.432025645	0
PAK3	5.539728734	0
PAK7	4.687338552	0
SEMA4D	3.456588028	0
ADAM23	2.299033785	0
EPHB2	2.728741378	0
SMO	0.422761137	0
BMP1	2.389216627	0
ADAM11	4.631441635	0
DPYSL2	2.284545748	0
LINGO1	2.15434803	0
ADAMTS7	2.484673007	0
EPHB4	0.273709611	0
TUBB4A	2.125011035	0
RTN4	2.057498911	0
PLXND1	2.284524635	0
DOCK1	0.465017957	0

PRKAR2B	2.486517642	0
TUBB2B	2.004743045	0
ABLIM3	0.358447897	0
GLI3	0.243223242	0
ROBO2	2.405882747	0
MIR23B	0.428406121	0
ITGA4	2.258284631	0

Supplementary Table 6a: Univariable cox regression analysis of survival by increasing *SEMA4D* expression across Group 3 and 4 medulloblastoma

Group	HR	95% CI	p-value
Group 3 and 4 (n=377)	0.5987	0.4538-0.7898	0.000283
Group 3 (n=113)	0.8996	0.58-1.395	0.636
Group 4 (n=264)	0.4622	0.2644-0.808	0.00676

Supplementary Table 6b: Statistical analyses of survival in patients exhibiting high *SEMA6A NRP1, NRP2, or L1CAM* gene expression relative to patients with low *SEMA6A, NRP1, NRP2 or L1CAM* gene expression

Gene Assignment	Survival_Pvalue	Survival_Qvalue
SEMA6A	0.107	0.433
NRP1	0.935	0.973
NRP2	0.419	0.723
L1CAM	0.961	0.981

Supplementary Table 7: Gene Set Enrichment Analysis (GSEA) results for Reactome and KEGG databases identified pathways significantly enriched in gene sets that were downregulated in D283 Scramble relative to OTX2 KD tumorspheres

NAME (Reactome Database)	SIZE	ES	NES	NOM p-val	FDR q-val	FWER p-val
SEMAPHORIN INTERACTIONS	63	-0.65	-1.96	0	0.0411	0.047
AXON GUIDANCE	237	-0.52	-1.88	0	0.0640	0.139
DEVELOPMENTAL BIOLOGY	377	-0.49	-1.84	0	0.0780	0.237
INTERACTION BETWEEN L1 AND ANKYRINS	21	-0.74	-1.84	0.0026	0.0591	0.240
SIGNALING BY ROBO RECEPTOR	28	-0.71	-1.81	0	0.0661	0.315
L1CAM INTERACTIONS	82	-0.52	-1.65	0.0030	0.3009	0.894
NETRIN1 SIGNALING	37	-0.58	-1.63	0.0078	0.3097	0.926
BOTULINUM NEUROTOXICITY	17	-0.67	-1.57	0.0300	0.4194	0.982
INSULIN SYNTHESIS AND PROCESSING	19	-0.65	-1.54	0.0389	0.4923	0.995
SEMA4D IN SEMAPHORIN SIGNALING	29	-0.59	-1.53	0.0354	0.4746	0.995
**NAME (KEGG Database)**	**SIZE**	**ES**	**NES**	**NOM p-val**	**FDR q-val**	**FWER p-val**
AXON GUIDANCE	126	-0.66	-2.21	0	0	0
ERBB SIGNALING PATHWAY	86	-0.51	-1.57	0.0185	0.5618	0.641
VASOPRESSIN REGULATED WATER REABSORPTION	43	-0.53	-1.48	0.0256	0.7283	0.873
CELL ADHESION MOLECULES CAMS	125	-0.43	-1.45	0.0101	0.6949	0.926
MAPK SIGNALING PATHWAY	260	-0.40	-1.43	0.0046	0.6085	0.938
TYPE I DIABETES MELLITUS	36	-0.52	-1.41	0.0635	0.5669	0.956
LONG TERM DEPRESSION	66	-0.46	-1.40	0.0483	0.5385	0.969
LONG TERM POTENTIATION	70	-0.46	-1.38	0.0718	0.5132	0.979
TYPE II DIABETES MELLITUS	46	-0.48	-1.37	0.0507	0.5142	0.987
T CELL RECEPTOR SIGNALING PATHWAY	105	-0.42	-1.36	0.0401	0.4860	0.988

Supplementary Table 8: GSEA revealed that genes associated with SEMA4D signaling were enriched in gene sets that were downregulated in D283 Scramble vs. OTX2 KD tumorspheres

GENE SYMBOL	GENE_TITLE	RUNNING ES
ARHGEF12	Rho guanine nucleotide exchange factor (GEF) 12	-0.5662338
ROCK2	Rho-associated, coiled-coil containing protein kinase 2	-0.54986334
ROCK1	Rho-associated, coiled-coil containing protein kinase 1	-0.55364746
MYL6	myosin, light chain 6, alkali, smooth muscle and non-muscle	-0.54413474
MYL12B	null	-0.5143689
MYH10	myosin, heavy chain 10, non-muscle	-0.48504782
PLXNB1	plexin B1	-0.4504612
MYH9	myosin, heavy chain 9, non-muscle	-0.40517303
MYL9	myosin, light chain 9, regulatory	-0.3621211
ARHGEF11	Rho guanine nucleotide exchange factor (GEF) 11	-0.29258606
SEMA4D	sema domain, immunoglobulin domain (Ig), transmembrane domain (TM) and short cytoplasmic domain, (semaphorin) 4D	-0.19715172
RHOB	ras homolog gene family, member B	-0.098245345
ARHGAP35	null	0.011831163
